# Adult Patients with Cancer Have Impaired Humoral Responses to Complete and Booster COVID-19 Vaccination, Especially Those with Hematologic Cancer on Active Treatment: A Systematic Review and Meta-Analysis

**DOI:** 10.3390/cancers15082266

**Published:** 2023-04-12

**Authors:** Efstathia Liatsou, Ioannis Ntanasis-Stathopoulos, Stavros Lykos, Anastasios Ntanasis-Stathopoulos, Maria Gavriatopoulou, Theodora Psaltopoulou, Theodoros N. Sergentanis, Evangelos Terpos

**Affiliations:** 1 Department of Clinical Therapeutics, National and Kapodistrian University of Athens, 11528 Athens, Greece; 2 Department of Public Health Policy, School of Public Health, University of West Attica, 12243 Aigaleo, Greece

**Keywords:** COVID-19, SARS-CoV-2, vaccination, booster, humoral response, antibodies, cancer, solid tumor, hematological malignancies

## Abstract

**Simple Summary:**

Taking into consideration the high risk of patients with cancer for severe COVID-19 infection, prioritization has been given to primary prevention with both primary and booster vaccination. However, robust evidence for vaccination efficacy remains limited, due to the lack of available clinical trials including patients with active cancer. The rates of both humoral and cellular immune response remain rather vague, and they are mainly based on data deriving from retrospective studies of limited internal and external validity. We aimed to gather and analyze the current available literature on the efficacy of COVID-19 vaccination among patients with different types of malignancies receiving different treatments. Our results highlight that patients with cancer present suboptimal immune responses after COVID-19 vaccination, which is more prominent among patients with hematological malignancies.

**Abstract:**

The exclusion of patients with cancer in clinical trials evaluating COVID-19 vaccine efficacy and safety, in combination with the high rate of severe infections, highlights the need for optimizing vaccination strategies. The aim of this study was to perform a systematic review and meta-analysis of the published available data from prospective and retrospective cohort studies that included patients with either solid or hematological malignancies according to the PRISMA Guidelines. A literature search was performed in the following databases: Medline (Pubmed), Scopus, Clinicaltrials.gov, EMBASE, CENTRAL and Google Scholar. Overall, 70 studies were included for the first and second vaccine dose and 60 studies for the third dose. The Effect Size (ES) of the seroconversion rate after the first dose was 0.41 (95%CI: 0.33–0.50) for hematological malignancies and 0.56 (95%CI: 0.47–0.64) for solid tumors. The seroconversion rates after the second dose were 0.62 (95%CI: 0.57–0.67) for hematological malignancies and 0.88 (95%CI: 0.82–0.93) for solid tumors. After the third dose, the ES for seroconversion was estimated at 0.63 (95%CI: 0.54–0.72) for hematological cancer and 0.88 (95%CI: 0.75–0.97) for solid tumors. A subgroup analysis was performed to evaluate potential factors affecting immune response. Production of anti-SARS-CoV-2 antibodies was found to be more affected in patients with hematological malignancies, which was attributed to the type of malignancy and treatment with monoclonal antibodies according to the subgroup analyses. Overall, this study highlights that patients with cancer present suboptimal humoral responses after COVID-19 vaccination. Several factors including timing of vaccination in relevance with active therapy, type of therapy, and type of cancer should be considered throughout the immunization process.

## 1. Introduction

The COVID-19 pandemic, declared on 1 March 2020, is responsible for 6,630,000 deaths worldwide [1,2]. The overall fatality rate has been reported to be 3.3%, with a particularly high disease-specific mortality risk for patients with cancer reaching 35–43% [1]. The relatively higher transmission rate and associated greater risk of mortality highlighted the urgent demand for efficient preventative vaccination [3]. Clinical trials were designed and held in less than a year, and the BNT162b2 COVID-19 vaccine was the first to receive emergency approval from the US Food and Drug Administration (FDA), followed by mRNA-1273, Ad26.COV2.S, AZD1222, and BBIP-CorV [4]. Based both on the findings of phase III clinical trials and real-world data, the efficacy of all these vaccines has been shown irrespectively of the severity of the disease [4].

Studies have increasingly reported on the outcomes of cancer patients, highlighting severe events such as intensive care unit admission, intubation, or death following COVID-19 infection [5]. Despite improvements in current therapies and medical care, rates have been found to be higher for patients with hematological malignancies, due to their systemic immunosuppressive state caused by the malignancy itself and the systematic therapy, pre-existing comorbidities, and frequent hospitalizations [6]. In addition to preventive contaminating measures such as personal hygiene and masks, vaccinations for such vulnerable populations were prioritized [5]. However, patients with malignancies were excluded from clinical vaccine trials, whereas most data derive solely from observational studies with a limited sample size [4]. On top of that, the impaired immune system raises concerns regarding the adequate production of SARS-CoV-2-specific antibodies post vaccination [7]. Booster vaccination has been shown to restore and sustain humoral response in healthy individuals [8,9,10]. It has become common practice to offer immunocompromised patients with cancer booster vaccinations to improve SARS-CoV-2 immunity to levels obtained in healthy individuals after the standard vaccination schedule, but pertinent data are scarce [11].

The aim of this systematic review and meta-analysis is to assess the rate of seropositivity in patients with hematological and solid cancers who have been vaccinated against COVID-19 and investigate any demographic or clinical factors that might affect immune response.

## 2. Methods

In this study, the updated Preferred Reporting Items for Systematic Reviews and Meta-analyses (PRISMA) reporting guideline was applied (Supplementary Materials, PRISMA_2020_checklist) [12].This Systematic Review doesn’t need a registration number of the database.

### 2.1. Search Strategy

**Figure 1:**
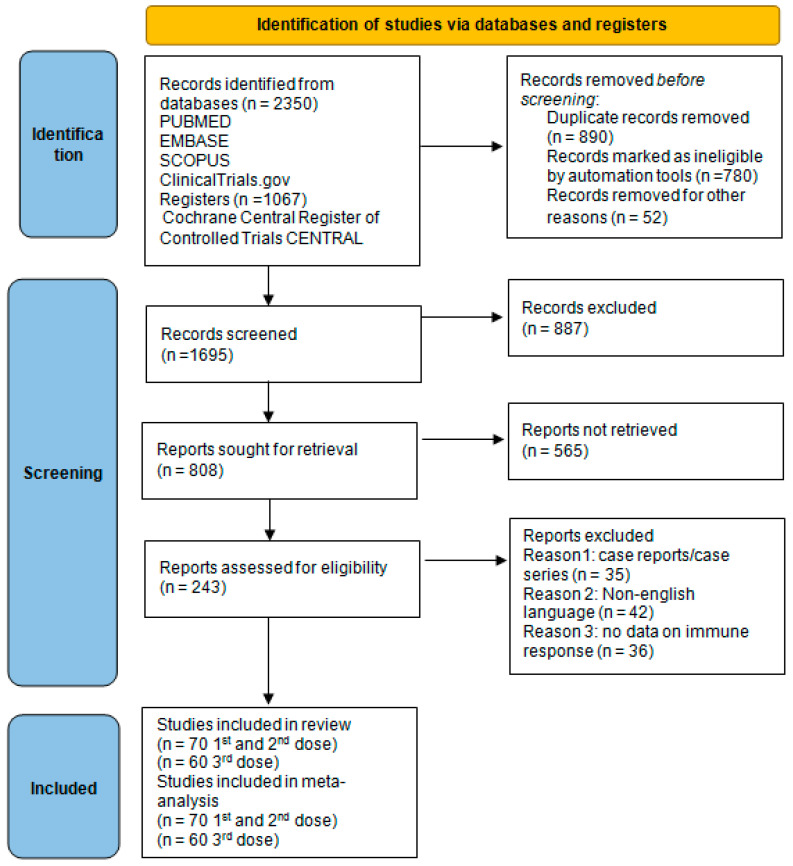
Flow chart.

We searched the Pubmed (1966–2022), Scopus (2004–2022), Clinicaltrials.gov (2008–2022), EMBASE (1980–2022), Cochrane Central Register of Controlled Trials CENTRAL (1999–2022), and Google Scholar (2004–2022) databases in our primary search along with the reference lists of electronically retrieved full-text papers. The date of our last search was set at 30 September 2022. Our search strategy included the query terms, as follows: “TUMOR OR CANCER OR MALIGNANCY OR NEOPLASIA OR LEUKEMIA OR LYMPHOMA OR SARCOMA OR NEOPLASM OR MYELOMA) AND (COVID-19 OR SARS-COV-2) AND (VACCINE OR BNT162B2 OR AZD1222 OR MRNA1273) AND (ANTIBODIES OR IMMUNORESPONSE OR RESPONSE OR HUMORAL OR SEROCONVERSION OR SEROPOSITIVITY OR IMMUNOGENICITY)”, and is schematically presented in the PRISMA flow diagram (Figure 1).

### 2.2. Study Selection

The database searches were imported to the COVIDENCE Systematic Review and two investigators (EL and SL) reviewed the title and abstracts. The same two authors evaluated the selected articles following the inclusion and exclusion criteria. In the case of any disagreement, a third author (INS) was included.

### 2.3. Study Selection

The criteria for the inclusion of studies were predetermined. Prospective or retrospective observational studies as well as randomized clinical trials that included adult patients with a diagnosis of hematologic or solid cancer after one, two or three doses of COVID-19 vaccine were considered for inclusion. Studies should have reported patients’ antibody response at specific time intervals. Case reports/series or cohort studies with an overall population of less than five patients were excluded. Studies with insufficient data on humoral response or data only on cellular response were also excluded.

### 2.4. Data Extraction

Data extraction was performed using Microsoft Office Excel. Extraction included the following items: (1) studies’ characteristics such as the title, digital object identifier (doi), date of publication, first author’s name, and study design; (2) demographic characteristics of patients including age, sex, history of SARS-CoV-2 infection, type of cancer, active therapy during vaccination, number of doses of vaccine received, the type of vaccine administrated, the time interval between vaccination, and the time point of antibodies evaluation; (3) the type of antibody that was evaluated and the methods applied for identification; and (4) the treatment scheme administered at the time of vaccination, time interval between therapy and vaccination, and the number of participants who were seropositive following immunization based on each treatment. The extracted data were double-checked and validated by two authors (EL and SL). A third author (INS) participated in team consensus in case of discrepancies.

### 2.5. Outcome Measures

The primary outcome was the humoral response of patients post vaccination in the form of the antibodies’ seropositivity rate as calculated by each study. The secondary outcomes included the rate of seroconversion after immunization according to the disease subtypes, treatment categories, and vaccine type.

### 2.6. Quality and Risk of Bias Assessment

The risk of bias and methodological quality of the included studies was evaluated independently by two authors (EL and SL) using the Newcastle-Ottawa Scale (NOS) (Tables S1 and S2), which evaluates the selection of the study groups, the comparability of the groups and the ascertainment of the exposure or outcome of interest [13]. A third author (INS) made the final decision on scoring in case of disagreement.

### 2.7. Statistical Analysis

A meta-analysis was performed using the STATA (version 2016). Dichotomous variables were assessed using the risk ratio (RR), continuous variables were assessed using the mean difference, and survival was assessed using the hazard ratio (HR). Statistical heterogeneity was assessed using the Higgins I2 statistic. The 95% confidence intervals (CI) were reported for all results. When mean values and standard deviations were not reported in the studies, values were calculated according to the equations proposed by Hozo et al. Moreover, the analyses were sub-grouped based on the cumulative dose of vaccination. Taking into account that the Ad26.COV2.s is a single shot vaccine, the emerged data related to this vaccine were analyzed in the second dose immunization group. Results were graphically displayed on forest plots. A qualitative analysis and demonstration of results was presented when the meta-analysis of the data was not feasible.

## 3. Results

### 3.1. Study Characteristics

Figure 1 shows the selection of studies. Overall, as regards the first and second vaccine shots, 70 studies were included in the meta-analysis [6,7,11,14,15,16,17,18,19,20,21,22,23,24,25,26,27,28,29,30,31,32,33,34,35,36,37,38,39,40,41,42,43,44,45,46,47,48,49,50,51,52,53,54,55,56,57,58,59,60,61,62,63,64,65,66,67,68,69,70,71,72,73,74,75,76]. In total, 10 studies [14,16,19,24,34,64,73,76,77,78] provided immune response information for patients with solid tumors, whereas 48 studies reported immune seroconversion results from patients with hematological malignancies [7,17,20,21,22,23,25,26,27,28,29,30,32,36,37,38,39,40,41,42,43,45,46,47,48,49,51,53,54,55,56,57,59,60,61,62,63,66,68,69,70,74,75,79,80,81,82], and 12studies [15,18,24,35,44,50,52,58,65,67,71,72] included patients with both solid and hematological malignancies. In total, 8 studies calculated patients’ antibodies after the first dose of vaccination only [16,19,26,27,31,41,51,62], whereas 35 studies evaluated immunogenicity after both doses [7,14,17,20,24,25,29,30,32,34,36,40,46,47,48,50,56,57,58,63,75,76,77,78,80,81] and 27 after the second dose only [15,18,21,22,23,25,28,35,37,38,39,40,42,43,44,45,46,47,52,53,54,55,59,61,63,64,65,66,67,68,69,70,71,72,73,74,80,82]. Studies were also stratified according to the type of vaccine that patients received. Of the 70 included studies, 30 studies reported vaccination results after vaccination with BN162b2 only [14,16,18,20,22,23,24,27,28,30,34,35,37,38,39,40,43,45,47,53,57,58,63,66,77,78,80,81,82,83], 20 studies reported results from vaccination with either BNT162b2 or mRNA-1273 vaccine [7,17,25,36,40,44,46,48,52,54,59,60,62,67,68,70,72,73,74,76], 11 studies reported results from vaccination with either BNT162b2 or AZD1222 vaccine [21,25,26,29,31,32,41,50,51,56,75], 6 studies included patients that received either BNT162b2 or AD26.COV2.S or mRNA-1273 [15,42,49,61,69,71], and 3 studies had available results from vaccination with BNT162b2 or AZD122 or mRNA-1273 [19,64,65]. One study reported results on mRNA-1273 exclusively [55].

### 3.2. First Vaccine Dose Immune Response

**Figure 2:**
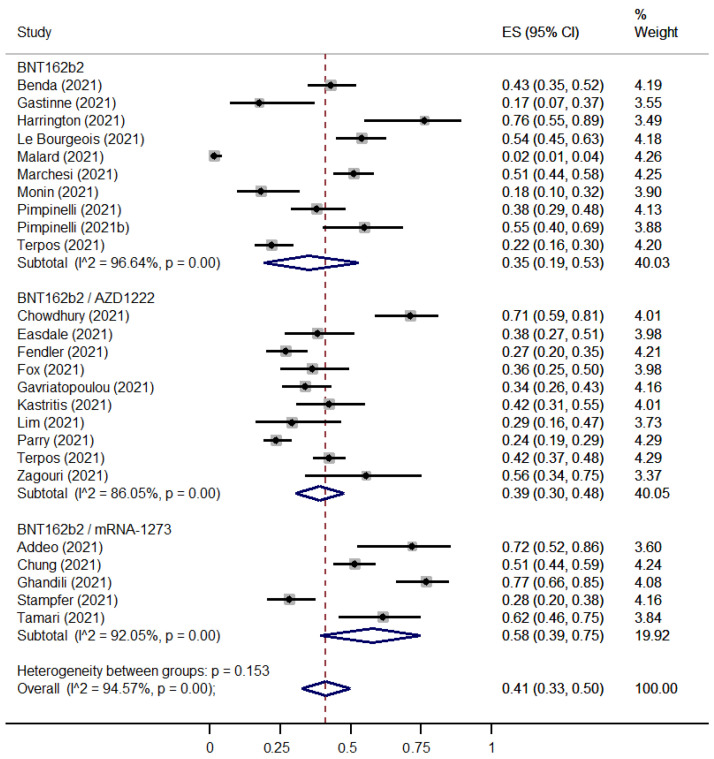
Pooled EffectSize (ES) for the immune seroconversion rates of patients with hematological malignancies after the first dose of COVID-19 vaccine. [7,19,20,25,26,27,29,30,31,36,38,40,41,48,50,51,56,57,62,63,66,75,80].

**Figure 3:**
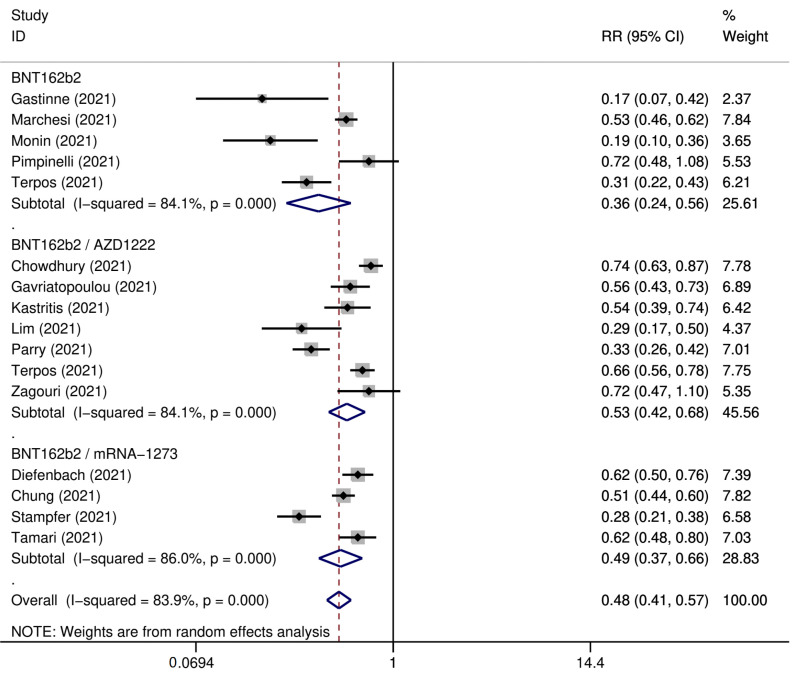
Pooled EffectSize (ES) for the immune seroconversion rates of patients with solid tumor after the first dose of COVID-19 vaccine [17,19,24,26,29,31,36,40,48,51,56,63,76,81].

Figure 2 and Figure 3 present the seroconversion rates for seroconversion among immunocompromised patients, with either hematological malignancies or solid tumors compared with immunocompetent controls after a first dose of COVID-19 vaccine, respectively. Data were available from a total of 2443 patients with hematological malignancies, 2079 patients with solid tumors and 239 immunocompetent controls who were vaccinated with the first vaccine shot against SARS-COV-2.

The rate of immune seroconversion for patients with hematologic cancer was 0.41 [95% CI: 0.33–0.50] (Figure 2), and for patients with solid tumors it was 0.56 [95% CI: 0.47–0.64] (Figure 3). The immunocompetent controls showed an immune response rate of 90% (Effect Size (ES): 0.90 [95% CI: 0.82–0.96]) (Figure S1). Risk Ratios were lower for patients with hematological malignancies (RR: 0.48 [95% CI: 0.41–0.57]) (Figure S2) in comparison with patients with solid tumors (RR: 0.57 [95% CI: 0.49–0.67]) (Figure S3).

### 3.3. Second Vaccine Dose Immune Response

Figures S4 and S5 present the percentages and risk ratios for seroconversion among immunocompromised patients compared with healthy controls following the second dose of vaccination after studies were stratified based on the type of vaccine. In total, 51 studies investigated response after mRNA vaccines (either BNT162b2 or mRNA-1273). A total of 8276 patients with hematological malignancies, 2230 patients with solid cancer, and 2494 controls were analyzed regarding seroconversion after the second dose against COVID-19.

**Figure 4:**
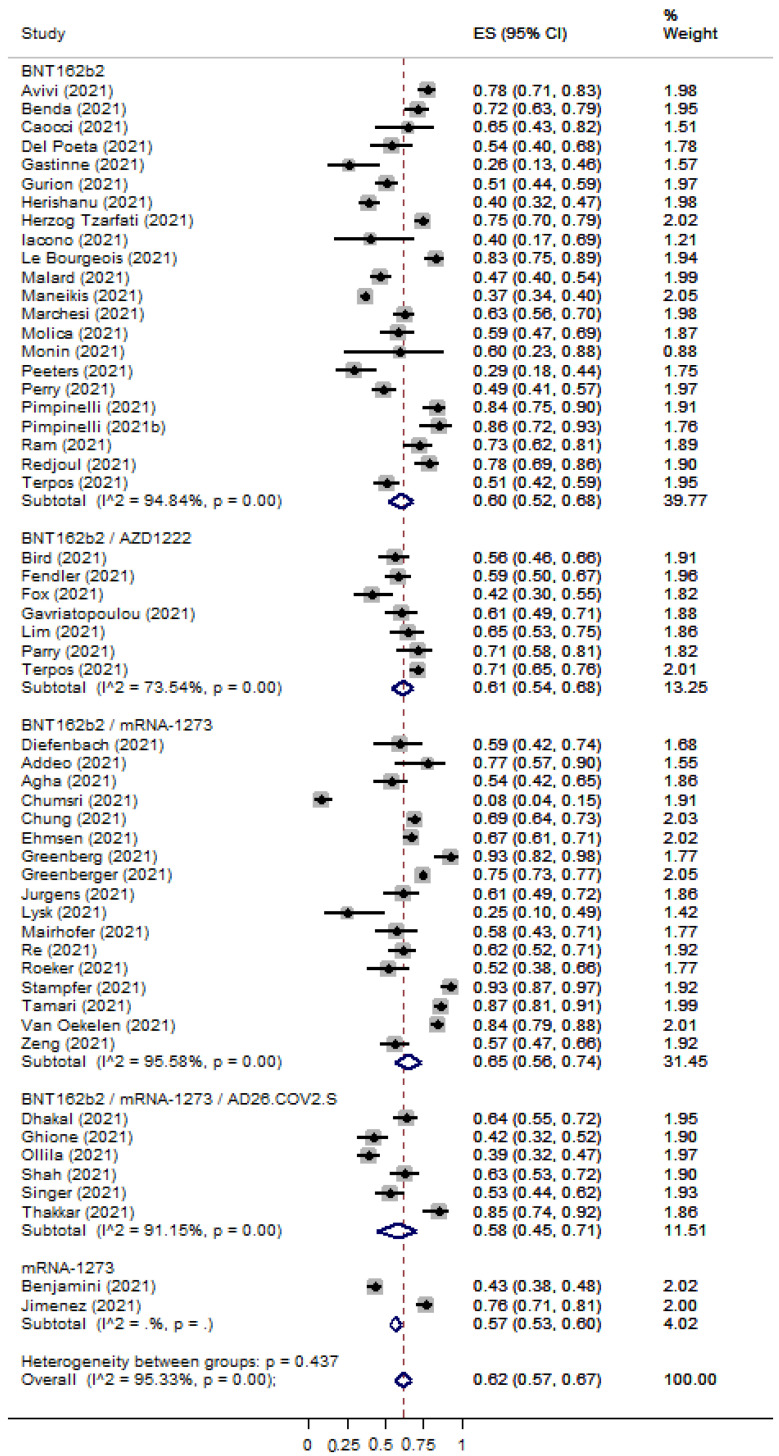
Pooled EffectSize (ES) for the immune seroconversion rates of patients with hematological malignancies after the second dose of COVID-19 vaccine. [7,8,15,17,18,20,21,22,23,24,25,28,29,32,35,36,37,38,39,40,42,43,44,45,46,47,48,49,50,52,53,54,55,56,57,58,59,60,61,63,66,67,68,69,70,71,74,75,80,81,82,84].

**Figure 5:**
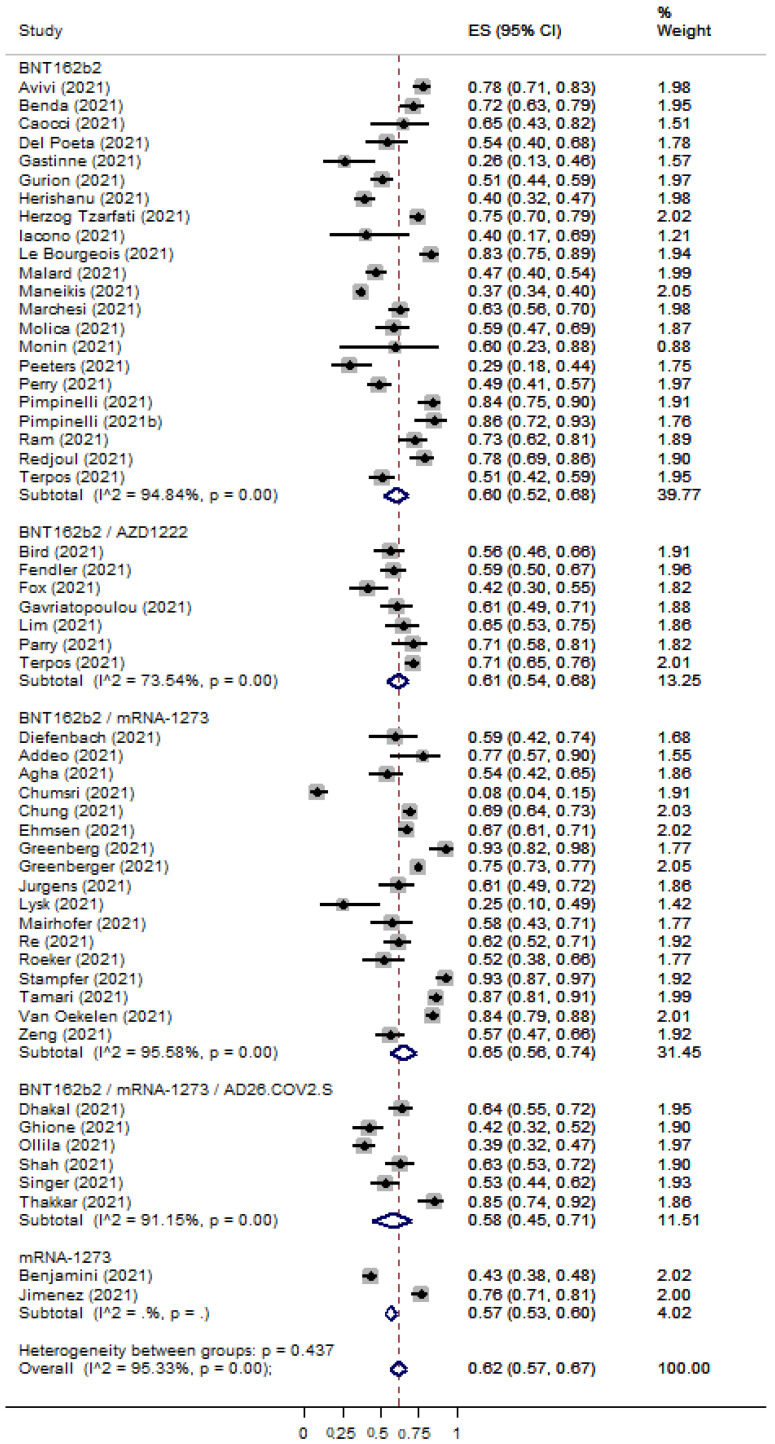
Pooled EffectSize (ES) for the immune seroconversion rates of patients with solid tumors after the second dose of COVID-19 vaccine. [7,15,17,18,20,22,23,24,25,28,35,36,37,38,39,40,40,42,43,44,45,46,47,48,49,52,53,54,55,57,58,59,60,61,63,66,67,68,69,71,72,74,80,81,82,85,86].

The overall percentage of immune seroconversion for patients with hematological malignancies was 0.62 [95% CI: 0.57–0.67] (Figure 4) and 0.88 [95% CI: 0.82–0.93] (Figure 5) for patients with solid tumors, respectively. The immunocompetent controls showed an immune response rate of 90% (ES: 0.90 [95% CI: 0.82–0.96]) (Figure S4). Risk Ratios were lower for patients with hematological malignancies (RR: 0.59 [95% CI: 0.53–0.63]) (Figure S5) in comparison with patients with solid tumors (RR: 0.85 [95% CI: 0.78–0.92]) (Figure S6).

### 3.4. Subgroup Analyses on Predictive Factors for Seroconversion after Initial Complete Vaccination

The analysis of the antibody response in patients with hematological malignancies and solid tumors was further applied based on potential predictive factors such as the type of disease, active or inactive treatment, type and time intervals of active treatment, and the type of vaccine that was administered. In terms of the subtype of hematological malignancy, data were available from 4 studies with 522 patients with multiple myeloma who received one vaccine dose (ES: 0.38 [95% CI: 0.20–0.59]) (Figure S7), and 17 studies encompassing data from 1814 patients with multiple myeloma who were fully vaccinated against SARS-COV-2 (ES: 0.80 [95% CI: 0.73–0.86]) (Figure S8). Furthermore, 1023 out of 1912 patients with chronic lymphocytic leukemia responded to full COVID-19 vaccination (ES: 0.51 [95% CI: 0.44–0.58]) (Figure S9). For Hodgkin lymphoma, 110 out of 115 patients showed immune seroconversion after the second dose of the COVID-19 vaccine, and the pooled response was ES: 0.99 [95% CI: 0.94–1.00]) (Figure S10). For non-Hodgkin lymphoma, immune response was evaluated in 10 studies with a total of 934 patients who completed vaccination with a seroconversion rate of 556/934 and a pooled ES of 60% (95% CI: 49%–71%) (Figure S11). Regarding immune response in patients with myelofibrosis, data were available in three studies; 22 out of 36 fully vaccinated patients showed adequate immune response and the pooled ES was 0.61 (95% CI: 0.44–0.78) (Figure S12). Finally, 321 out of 402 patients with myelodysplastic and myeloproliferative syndromes demonstrated an immune response after full COVID-19 vaccination, with a pooled ES of 80% [95%CI: 69%–90%] (Figure S13).

We also analyzed available data on seroconversion based on the treatment status of patients. A significant difference in antibody response was found for active treatment at the time of vaccination in comparison with no treatment. More specifically, 1392 out of 2395 patients under active treatment and 1000 out of 1381 patients without treatment showed an antibody response, and the pooled responses were 48% [95% CI: 36%–61%] and 76% [95% CI: 67%–83%] for active treatment and no treatment, respectively (Figures S14 and S15, respectively). The RR was calculated from 9 studies for active treatment and 11 studies for no treatment, and immune response in cancer patients was compared with antibodies measurement in healthy controls after full vaccination. The pooled RR was 0.49 [95% CI: 0.40–0.59] for active treatment and 0.79 [95% CI: 0.70–0.8] for no treatment (Figures S16 and S17, respectively).

Furthermore, different types of treatment were evaluated. The lowest antibody titers following full immunization were observed in patients on active therapy with anti-CD20 antibodies; only 43 out of 426 patients (ES: 11% [95% CI: 0.5%–20%]) achieved detectable antibody titers (Figure S18). The RR for anti-CD20 treatment was 0.16 [95% CI: 0.09–0.28] as pooled from four studies (Figure S19). A subgroup analysis of 412 patients revealed that the time interval between anti-CD20 therapy and vaccination influenced immune response (ES: 0.60 [95% CI: 0.47–0.72], RR: 0.78 [95% CI: 0.66–0.91]) (Figures S20 and S21, respectively).Beside anti-CD20 therapy, low seropositivity rates after full vaccination were evident for those who received Bruton’s kinase inhibitors as shown in 14 studies (*n* = 244/691, ES: 33% [95% CI: 20%–48%], RR: 0.22 [95% CI: 0.13–0.37], ref: healthy controls) (Figures S22 and S23, respectively) and CAR-T cell therapy (*n* = 43/104, ES: 38% [95% CI: 22–56%], RR: 0.44 [95% CI: 0.22–0.88]) (Figures S24 and S25, respectively). Conversely, the highest proportion of antibody response among the patients on treatment was estimated for patients who received chemotherapy (*n* = 601/806, ES: 72% [95% CI: 62%–81%]) (Figure S26) and endocrine therapy (*n* = 82/107, ES: 89% [95% CI: 37%–100%]) (Figure S27). Four studies included 104 patients who received combination treatment, out of whom 73 were seropositive (ES: 59% [95% CI: 32%–84%]) (Figure S28).

A total of 1402 patients were evaluated in nine studies involving hematopoietic stem cell transplantation. There was no difference between groups for allogeneic and autologous transplants. For allogeneic transplants, 627 out of 760 patients achieved an antibody response, and the pooled response was 82% [95% CI: 78%–87%] (Figure S29). Most patients underwent transplantation more than 1 year prior to vaccination. Limited data showed reduced response rates particularly for those receiving allogeneic transplantation less than 6 or 12 months prior to vaccination. For autologous transplants, 522 out of 642 patients achieved an antibody response, and the pooled response estimate was 78% [95% CI: 67%–88%] (Figures S30 and S31).

The immune response was also evaluated in a subgroup analysis based on the type of vaccine that was received. More specifically, most patients received the BNT162B2 vaccine; 1068 patients with hematological malignancies received only one dose of the vaccine, and 5885 patients got fully vaccinated. Among them, 522 and 3647 produced adequate antibody titers with an ES of 46% [95% CI: 35%–57%] and 63% [95% CI: 56%–69%], after one and two vaccine shots, respectively (Figures S32 and S33, respectively). Immunogenicity after BNT162B2 vaccination was also investigated in patients with solid tumors, with 619 out of 1058 and 916 out of 976 developing adequate immune responses after the first and second doses, respectively (ES: 56% [95% CI: 48%–63%] and 94% [95% CI: 93%–96%]) (Figures S34 and S35, respectively). Seroconversion rates were also calculated in 11 studies including 1596 patients with hematological malignancies who were vaccinated with mRNA-1273. The ES was found to be 0.72 [95% CI: 0.57–0.85] (Figure S36). Lastly, three studies reported on full vaccination with the AZD1222 of 89 patients with hematological malignancies, 47 of whom produced a sufficient amount of antibodies with a pooled ES of 47% [95% CI: 24%–71%] (Figure S37).

### 3.5. Booster COVID-19 Vaccination

In total, 60 studies were included for data analysis on booster COVID-19 vaccination, 43 studies reported on the immune response of a total of 4754 patients with a diagnosis of hematological malignancy, and 22 studies determined immune seroconversion in 2440 patients with solid tumors [78,79,84,85,86,87,88,89,90,91,92,93,94,95,96,97,98,99,100,101,102,103,104,105,106,107,108,109,110,111,112,113,114,115,116,117,118,119,120,121,122,123,124,125,126,127,128,129,130,131,132,133]. The median age of cancer patients was 64 years (range: 43–76). Studies were grouped according to the type of vaccine that was administered as a booster dose. The mean time of evaluation following booster vaccination was 28 days (range: 7–95).According to the studies that had available information on the specific type of hematological malignancy or solid tumor, 109 patients had ALL, 100 AML, 70 CML, 1003 CLL, 755 lymphomas, 64 MPN/MDS, 11 PV, 32 ET, 792 MM, 121 WM, 407 breast cancer, 281 urological cancer, 95 gynecological cancer, 92 melanoma, 347 lung cancer, 327 GI cancer, 19 brain cancer, 65 head and neck cancer, and 9 connective tissue cancer. Data regarding treatment status was available in 41 studies. More specifically, 3991 patients were in active treatment, and 1278 were off treatment.

**Figure 6:**
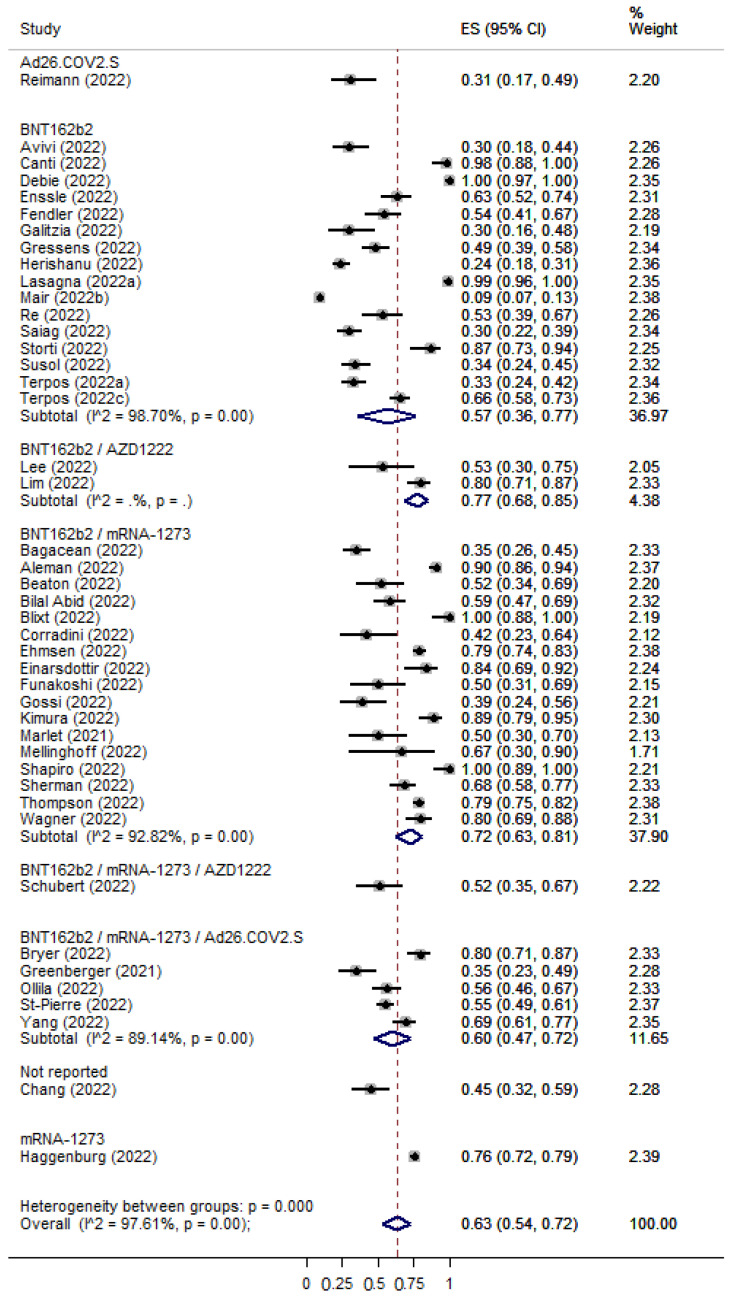
Pooled EffectSize (ES) for the immune seroconversion rates of patients with hematological malignancies after the third dose of COVID-19 vaccine [28,74,85,87,88,89,91,92,93,95,96,97,98,99,100,101,104,105,107,108,109,110,111,112,113,114,114,116,117,118,122,123,124,125,126,127,128,129,130,131,132,134,135].

**Figure 7:**
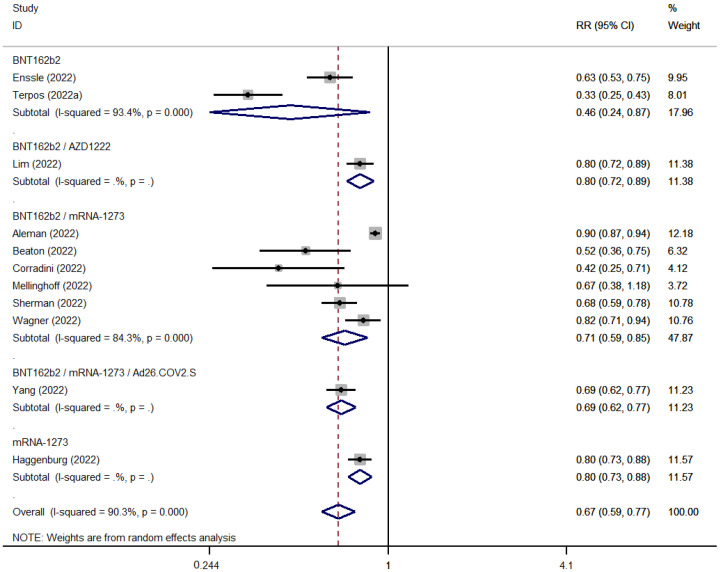
Pooled RelativeRisk (RR) for the immune seroconversion of patients with hematological malignancies after the third dose of COVID-19 vaccine [95,99,103,107,108,109,110,116,124,127,131].

**Figure 8:**
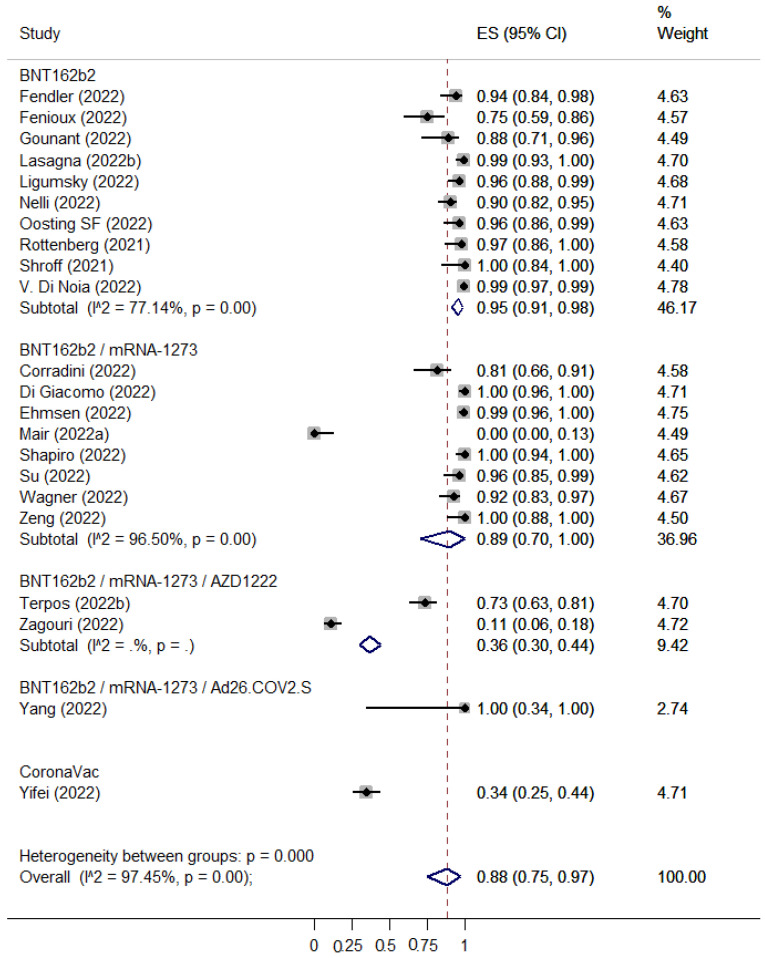
Pooled EffectSize (ES) for the immune seroconversion rates of patients with solid tumors after the third dose of COVID-19 vaccine. [33,67,77,78,79,87,90,92,99,102,106,109,112,114,115,119,120,127,129,132,133,135].

**Figure 9:**
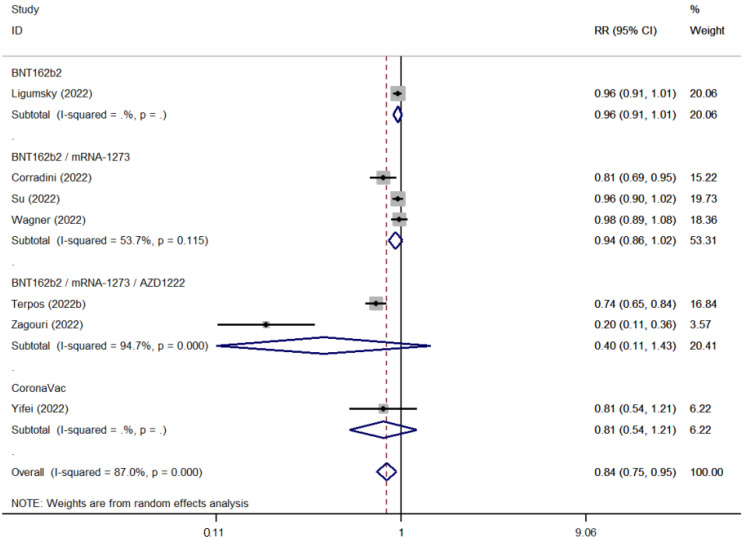
Pooled RelativeRisk (RR) for the immune seroconversion of patients with solid tumors after the third dose of COVID-19 vaccine [79,99,106,109,127,129,135].

SARS-COV-2 booster vaccination showed reduced seroconversion rates in patients with hematological malignancies as compared to healthy controls (ES: 0.63 [95% CI: 0.54–0.72] versus ES: 0.98 [95% CI: 0.89–1.00]) (Figure 6 and Figure 7). The pooled seropositivity RR was also lower for patients with hematological malignancies (RR: 0.67 [95% CI: 0.59–0.77]) compared to the pooled rates for patients with solid tumors (RR: 0.84 [95% CI: 0.75–0.95]) (Figure 8 and Figure 9).

### 3.6. Subgroup Analyses on Predictive Factors for Seroconversion after Booster Vaccination

We then divided patients into subgroups based on the treatment they received. Patients undergoing active therapy with anti-CD20 Abs showed the lowest seroconversion rate following booster immunization; only 26% [95% CI: 16–37%] of 442 patients achieved detectable antibody titers (Figure S38). Beside anti-CD20 therapy, the lowest seropositivity rate after booster vaccination was evident for those who received combination therapy of two or more therapeutic agents (*n* = 404, ES 45% [95% CI: 25%–65%]) (Figure S39) and CAR-T cell therapy (*n* = 133, ES 32% [95% CI: 22–43%], I2 = 85.1%) (Figure S40). In contrast, the highest proportion of antibody response was estimated for patients who had undergone allogenous SCT (*n* = 164, ES 72% [95% CI: 47–92%]) (Figure S41) and steroids (*n* = 252, ES 71% [95% CI: 53–87%]) (Figure S42). Studies reporting on patients who received venetoclax showed mixed results (*n* = 85, ES 50% [95% CI: 25–74%]) (Figure S43). Following booster immunization, the low rates of achieving an antibody response were also observed for patients with hematological malignancies who were on Bruton’s tyrosine kinase (BTK) inhibitors (*n* = 404, ES 45% [95% CI: 25–65%]) (Figure S44), while high rates of immune response were observed with autologous SCT (*n* = 126, ES 65% [95% CI: 32–93%]) (Figure S45). High rates of heterogeneity should be noted in the subgroup analyses on treatment subtypes.

When comparing antibody responses in patients with different types of hematological malignancies and solid tumors, data were available only for subtypes of hematological cancer. The lowest seropositivity rate was evident in patients with non-Hodgkin lymphoma (*n* = 557, ES 48% [95% CI: 35–60%]) (Figure S46), followed by those with CLL (*n* = 1003, ES 65% [95% CI: 49–79%]) (Figure S47) and MM (*n*= 792, ES: 86% [95% CI: 77–94%]) (Figure S48). Regarding immune response rates by vaccine type, data wereavailable for pooled analysis only from studies with the BNT162B2 booster vaccination. The Effect Size for antibody response was ES: 0.69 [95% CI: 0.43–0.77] (Figure S49) for patients with hematological malignancies with RR: 0.67 [95% CI: 0.59–0.77] (Figure S50), whereas for patients with solid tumors, the pooled Effect Size was found to be 0.90 [95% CI: 0.77–0.98] (Figure S51).

## 4. Discussion

This study showed that oncology patients had a significantly reduced antibody response compared with healthy individuals following the first, second, and booster COVID-19 vaccination. More specifically, seroconversion was less likely in patients with blood cancer compared with healthy individuals by 52%, 41%, and 33% after the first, second, and booster vaccine shot, respectively. Seroconversion was also less likely in patients with solid cancer compared with healthy controls by 43%, 15%, and 16% after each vaccine dose, respectively. It has to be noted that the anti-SARS-CoV-2 humoral response was more attenuated in patients with hematological cancer compared to patients with solid tumors. Following complete vaccination, immune response was mostly affected in patients with CLL (46% lower seroconversion compared to controls) or NHL (38% lower seroconversion), in patients on active treatment (51% lower seroconversion), and in those receiving drugs targeting CD20 (84% lower seroconversion) or BTKIs (78% lower seroconversion) or CAR-T cell therapy (60% lower seroconversion). Among patients with cancer, those with solid tumors or MPN had the highest seroconversion rates after the first vaccine dose, whereas those with HL had the highest seroconversion rates after the second dose. Similar results were derived from the pooled analysis of the booster vaccine shot; hematological patients had 33% and patients with solid cancer had 16% lower seroconversion compared to healthy controls.

According to the pooled response rate analysis, the type of vaccine administered has an effect on antibody production with the mRNA-1273 vaccine being the most effective followed by the BNT162B2 and the AZD122.This is also supported by Noori et al., where lower seropositivity rates were observed with the BNT162B2 vaccination compared to mRNA-1273 (RR: 0.89, 95% CI: 0.79–0.99) [134,135,136].

These findings can be explained by both the underlying diseases and the therapeutic approaches that impair the immune response. This immune deregulation has also become evident in studies evaluating the clinical manifestations and outcomes of COVID-19-infected cancer patients [137]. First, patients with cancer, especially those with hematological malignancies, experience long periods of neutropenia not only due to the anticancer therapy they receive, such as anti-CD20 antibodies and HSCT, but also as a result of the malignancy’s natural course itself through immunological mechanisms or direct bone marrow infiltration [138]. The humoral adaptive immune response is affected on multiple levels [138,139]. Lymphopenia is commonly found, and in that case, B cells are highly depleted, principally in cases of CLL and MM [139]. Treatments targeting CD20, CD38, or B-cell maturation antigen (BCMA) have also been associated with depleted circulating B-cells and significantly impaired IgM and IgG responses against both the ancestral Wuhan strain and the Omicron SARS-CoV-2 variants [139,140,141]. The defect in humoral response may remain evident even after a second booster vaccine dose, as it has been shown in patients with MM on anti-BCMA treatment [8].

The cellular component of the immune responses also affected, as has been shown in studies that profiled the cytometric activity of patients with cancer and COVID-19 infection [142]. More specifically, a statistically significant difference was observed in CD4+ T-cells being less frequent in patients with hematologic cancer compared to those who suffer from solid malignancies, whereas CD8+ T-cells were equally detected among those groups [142]. Similar variations have been confirmed and are consistent with findings from other studies on vaccines against viruses (influenza, hepatitis B) and bacteria (Streptococcus pneumoniae) among patients with hematological malignancies and solid organ transplant recipients [143,144]. However, only a few studies provided detailed data regarding cellular response, and high levels of heterogeneity did not permit a pooled analysis [144].

The vaccination-induced response against SARS-CoV-2 in patients with cancer has been examined in other systematic reviews and meta-analyses as well. Gagelmann et al. presented results from a total of 49 studies including patients with hematological malignancies. The authors showed an impaired antibody production, which was mainly associated with the type of malignancy, with the lowest immune response being noted in cases of chronic lymphocytic leukemia, and the administration of active treatment at the time of vaccination [145]. The large sample of patients constitutes one of the main strengths of that study; however, patients with solid tumors were not included in the study protocol, a fact that could otherwise further enlighten potential tendencies and differences in a more direct way [145]. Another systematic review by Noori et al. investigated the effect of the two-dose vaccination scheme in antibody production, and the results are consistent with our findings in terms of the defect in the humoral response following COVID-19 vaccination, especially in patients with hematological cancer. Nevertheless, our study is still the most updated regarding the systematic analysis of both the two-dose complete vaccination scheme and booster vaccination in all patients with cancer.

Our study has some limitations to be acknowledged. First, considerable heterogeneity was observed in some subgroups that were analyzed due to the misrepresentation of these subpopulations. More specifically, studies with AML, CML, WM, MF, PV, IT, and CML had limited numeric data regarding serological response; thus, no pooled data were available. The effect of other potential confounding factors, including prior COVID-19 infection and time from last infection to last vaccination, age, body mass index, and autoimmune diseases, on our results cannot be excluded [146,147,148]. However, due to a lack of reported pertinent data, we were unable to include such data in our analyses. Under this framework, subgroup analysis included a pooled analysis of the humoral response in patients with solid tumors who received chemotherapy. There is an undoubtedly wide range of different chemotherapeutic agents in various administration forms and schemes with different bioavailability and toxicity profiles and a heterogenous degree of immune suppression. However, data on each patient’s therapeutic regimen scheme was unavailable, making further analysis unfeasible. Regarding patients on ruxolitinib or immune checkpoint inhibitors or cellular therapies, as well as those receiving radiotherapy, pooled analyses were not performed due to the small number of arms within each subgroup. Patients who underwent an allogeneic stem cell transplant constitute a specific clinical group selected based on predefined clinical criteria. Current approaches to prevent and treat GVHD post transplantation include a constellation of immunosuppressive medications. Therefore, this is a highly heterogenous group of patients and the findings of immune response to vaccination should be interpreted cautiously. Furthermore, different units of antibody titers and time intervals were assessed in the included studies, which resulted in significant heterogeneity in the pooled results. Additionally, the majority of studies evaluated the efficacy of either BNT162b2 or mRNA-1273; thus, the generalizability of the pooled results on non-mRNA vaccines may be negotiable. The antibody’s titers as a surrogate endpoint could reflect the efficacy of the immune system against COVID-19 [149,150]. However, different diagnostic assays were used by each study to evaluate the immune response post vaccination [151]. The anti-spike IgG antibody was the most commonly tested in studies; however, their neutralizing activity, and thus efficacy against infection, was not consistently reported. The absence of a standard-of-care assay to determine the humoral immune response post COVID-19 vaccination may limit the clinical utility of determining antibody response in the clinical practice [151]. Regarding the efficacy assessment post vaccination, the antibody cut-off to define seropositivity as well as the measurement units are not unanimous among studies.This high level of heterogeneity made a potential subgroup analysis unfeasible as multiple subgroups with low patient numbers were derived. In addition to the above, given that COVID-19 is a viral infection, the role of CD8+ T cells has been examined and found to be protective, and it may be even more important in patients with hematological malignancies who have impaired humoral responses [152,153].

## 5. Conclusions

In conclusion, our results show that patients with cancer have impaired humoral responses to complete and booster COVID-19 vaccination. This is more pronounced in patients with hematologic cancer on active treatment at the time of vaccination. The high level of heterogeneity in the methods and reported outcomes among the studies necessitates the careful evaluation of subgroup analyses. Patients with cancer should be prioritized for receiving booster and updated vaccine shots, pre- and post-exposure prophylaxis with antiviral drugs, and monoclonal antibodies in order to prevent severe COVID-19 outcomes.

## Data Availability

Data available upon reasonable request from the corresponding author.

## Supplementary Materials

The following supporting information can be downloaded at: https://www.mdpi.com/article/10.3390/cancers15082266/s1. Table S1. Newcastle-Ottawa Assessment Scale for cohort studies; Table S2. Newcastle-Ottawa Assessment Scale for case-control studies; Figure S1. Pooled Effect Size for immune seroconversion rates of immunocompetent controls after first vaccine dose COVID-19; Figure S2. Relative Risk for immune seroconversion of patients with hematological malignancies after first vaccine dose; Figure S3. Relative Risk for immune seroconversion of patients with solid tumors after first vaccine dose; Figure S4. Pooled Effect Size for immune seroconversion rate of immunocompetent controls after second vaccine dose; Figure S5. Relative Risk for immune response of patients with hematological malignancies after second vaccine dose; Figure S6. Relative Risk for immune response of patients with solid tumors after second vaccine dose; Figure S7. Effect Size for immune seroconversion rates in patients with multiple myeloma after first vaccine dose; Figure S8. Εffect size for immune seroconversion rates of patients with multiple myeloma after second vaccine dose; Figure S9. Effect size for immune seroconversion rates of patients with chronic lymphocytic leukemia after second vaccine dose; Figure S10. Effect size of immune seroconversion rates of patients with Hodgkin lymphoma after second vaccine dose; Figure S11. Effect size of immune seroconversion rates of patients with non-Hodgkin myeloma after second vaccine dose; Figure S12. Effect size of immune seroconversion rates of patients with myelofibrosis after second vaccine dose; Figure S13. Effect Size for immune seroconversion rates of patients with myelodysplastic and myeloproliferative syndromes after second vaccine dose; Figure S14. Effect Size for immune seroconversion rates of patients under active treatment after second vaccine dose; Figure S15. Effect Size for immune seroconversion rates of treatment naïve patients after second dose; Figure S16. Relative Risk for immune seroconversion rates of patients under active treatment after second vaccine dose; Figure S17. Relative Risk for immune seroconversion rates of treatment naïve patients after second vaccine dose; Figure S18. Effect Size for immune seroconversion rates of patients under active anti-CD20 monoclonal antibodies after second vaccine dose; Figure S19. Relative Risk of immune seroconversion rates of patients under active anti-CD20 monoclonal antibodies after second vaccine dose; Figure S20. Effect Size for immune seroconversion rates of patients having received treatment with anti-CD20 longer than one year ago; Figure S21. Relative Risk for immune seroconversion rates of patients having received treatment with anti-CD20 longer than one year ago; Figure S22. Effect Size of immune seroconversion rates of patients under active Bruton’s Tyrosine Kinase inhibitors treatment after second dose; Figure S23. Relative Risk of immune seroconversion rates of patients under active Bruton’s Tyrosine kinase inhibitors treatment after second dose; Figure S24. Effect Size for immune seroconversion rates of patients under CAR-T cells therapy after second vaccine dose; Figure S25. Relative Risk of immune seroconversion rate of patients under CAR-T cells therapy after second vaccine dose; Figure S26. Effect Size for immune seroconversion rates of patients under chemotherapy after second vaccine dose; Figure S27. Effect Size for immune seroconversion rates of patients under endocrine therapy after second vaccine dose; Figure S28. Effect Size for immune seroconversion rates of patients under combination treatment after second vaccine dose; Figure S29. Effect size for immune seroconversion rates of patients who underwent allogeneic stem cell transplantation after second vaccine dose; Figure S30. Effect Size for immune seroconversion rates of patients who underwent autologous stem cell transplantation after second vaccine dose; Figure S31. Relative Risk for immune seroconversion rates of patients who underwent allogeneic stem cell transplantation after second vaccine dose; Figure S32. Effect Size for immune seroconversion rates in patients with hematological malignancies who received first dose of BNT162B2 vaccine; Figure S33. Effect Size of immune seroconversion rates in patients with hematological malignancies who received second dose of BNT162B2; Figure S34. Effect Size for immune seroconversion rates of patients with solid tumors who received first dose of BNT162B2 vaccine; Figure S35. Effect Size for immune seroconversion rates of patients with solid tumors who received second dose of BNT162B2 vaccine; Figure S36. Effect Size for immune seroconversion rates of patients with hematological malignancies after second mRNA-1273; Figure S37. Effect Size for immune seroconversion rates of patients with hematological malignancies after second AZD1222; Figure S38. Effect Size for immune seroconversion rates of patients on active therapy with anti-CD20 monoclonal antibodies after third vaccine dose; Figure S39. Effect Size for immune seroconversion rates of patients on active therapy with combination therapy after third vaccine dose; Figure S40. Effect Size for immune seroconversion rates of patients under active CAR-T cell therapy after third vaccine dose; Figure S41. Effect Size for immune seroconversion rates of patients who underwent allogenic stem cell transplant after third vaccine dose; Figure S42. Effect Size for immune seroconversion rates of patients on treatment with steroids after third vaccine dose; Figure S43. Effect Size for immune seroconversion rates of patients under venetoclax therapy after third vaccine dose; Figure S44. Effect Size for immune seroconversion rates of patients on Bruton’s Tyrosine Kinase inhibitors after third vaccine dose; Figure S45. Effect size for immune seroconversion rates of patients who underwent autologous stem cell transplantation after third vaccine dose; Figure S46. Effect Size for immune seroconversion rates of patients with non-Hodgkin lymphoma after third vaccine dose; Figure S47. Effect size of immune seroconversion rates of patients with chronic lymphocytic leukemia after third vaccine dose; Figure S48. Effect Size of immune seroconversion rates of patients with multiple myeloma after third vaccine dose; Figure S49. Effect Size for immune seroconversion rates of patients with hematological malignancies who received BNT162B2 booster dose; Figure S50. Relative Risk of immune seroconversion rates of patients with hematological malignancies who received a BNT162B2 booster vaccine dose; Figure S51. Effect Size of immune seroconversion rates of patients with solid tumors who received a BNT162B2 booster vaccine dose. PRISMA_2020_checklist.

## Abbreviations

ALL	Acute Lymphocytic Leukemia
AML	Acute Myelocytic Leukemia
CML	Chronic Myelocytic Leukemia
CLL	Chronic Lymphocytic Leukemia
MPN/MDS	myeloproliferative neoplasms/myelodysplastic syndrome
PV	Polycythemia Vera
ET	Essential Thrombocytosis
MM	Multiple Myeloma
WM	Waldenström’s Macroglobulinemia
Gyn	Gynecological
GI	gastrointestinal
ES	Effect Size
RR	Relative Risk
SCT	Stem Cell Transplantation
