# Supplementary material for: Adult Patients with Cancer Have Impaired Humoral Responses to Complete and Booster COVID-19 Vaccination, Especially Those with Hematologic Cancer on Active Treatment: A Systematic Review and Meta-Analysis
Source: Cancers (Basel). 2023 Apr 12;15(8):2266. doi: 10.3390/cancers15082266 (PMC10136728; doi:10.3390/cancers15082266)
Supplement: Supplementary file 1 [file cancers-15-02266-s001.zip › cancers-2252272-Suppl Figures-done.pdf]

Supplemental documents

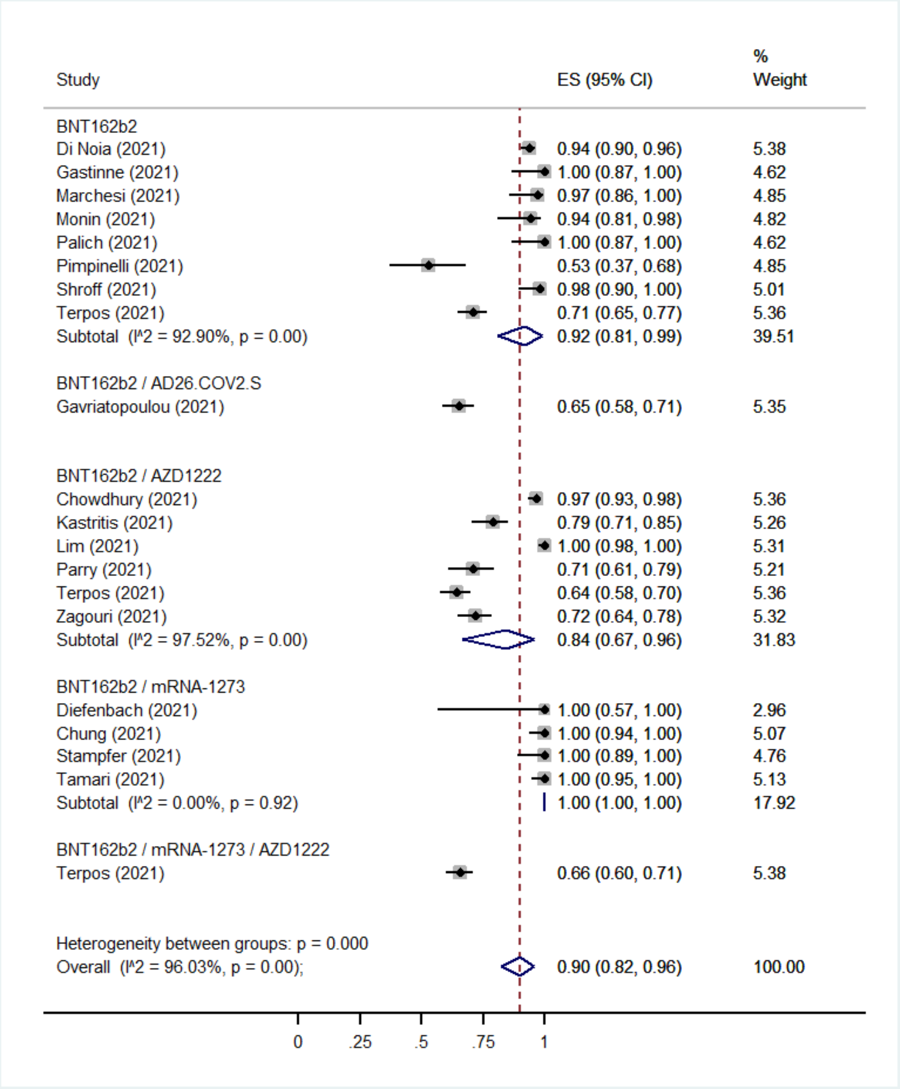

Figure S1. Pooled Effect Size for immune seroconversion rates of immunocompetent controls after first vaccine dose COVID-19.

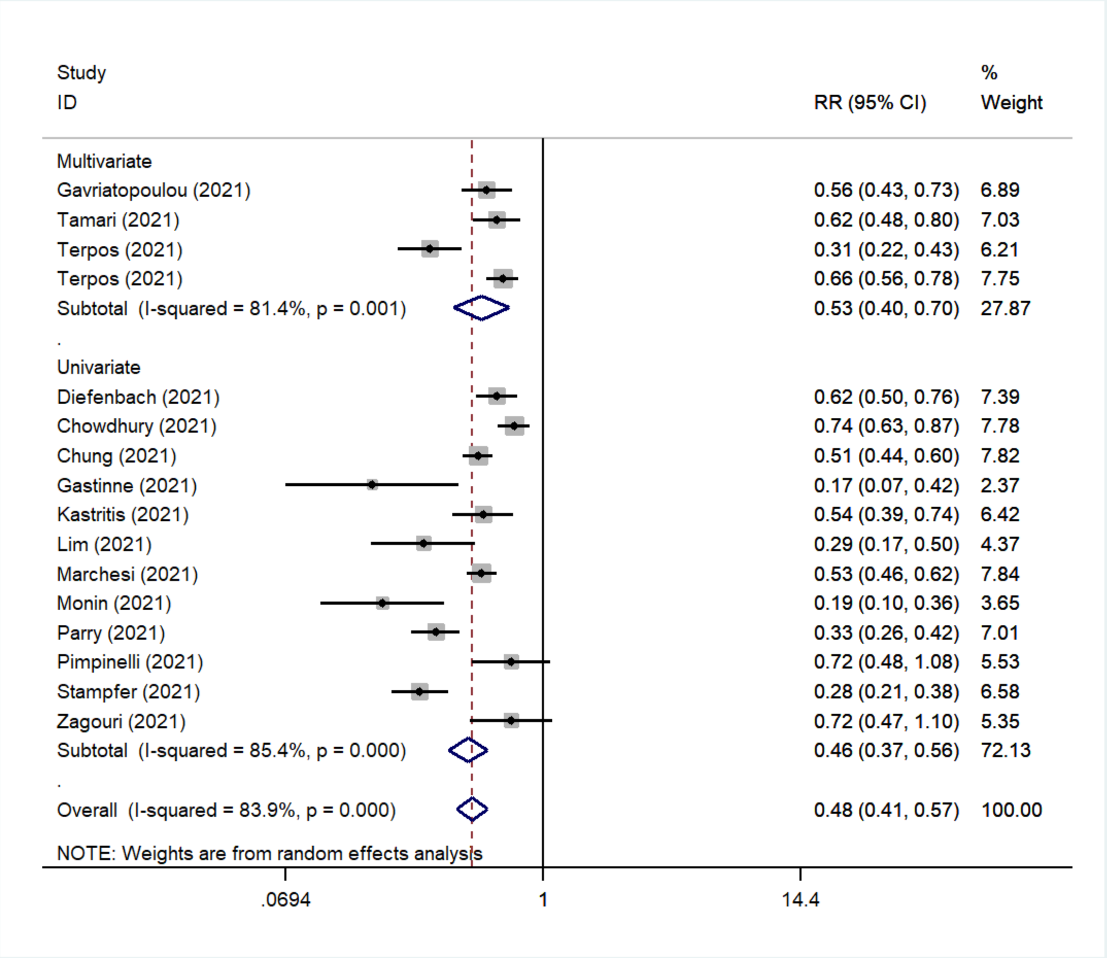

Figure S2. Relative Risk for immune seroconversion of patients with hematological malignancies after first vaccine dose.

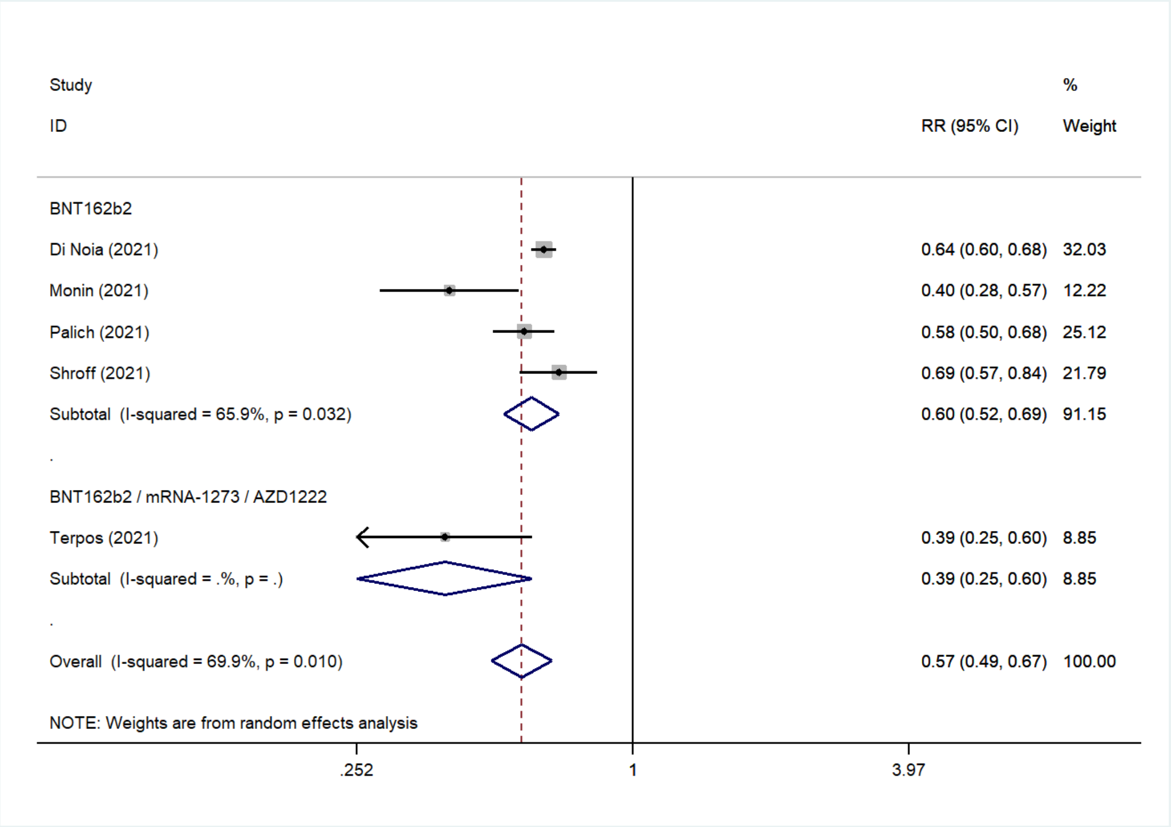

Figure S3. Relative Risk for immune seroconversion of patients with solid tumors after first vaccine dose.

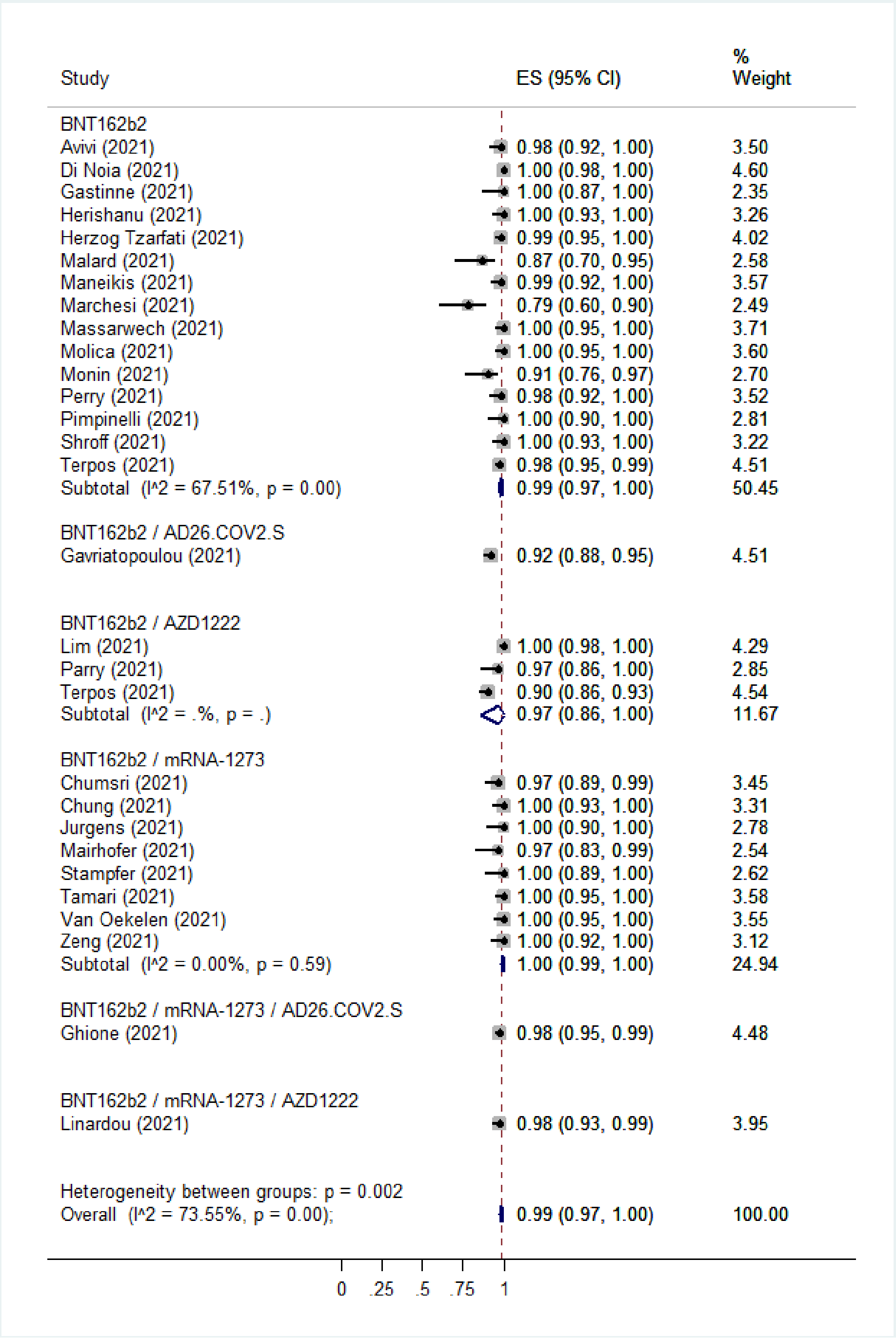

Figure S4. Pooled Effect Size for immune seroconversion rate of immunocompetent controls after second vaccine dose.

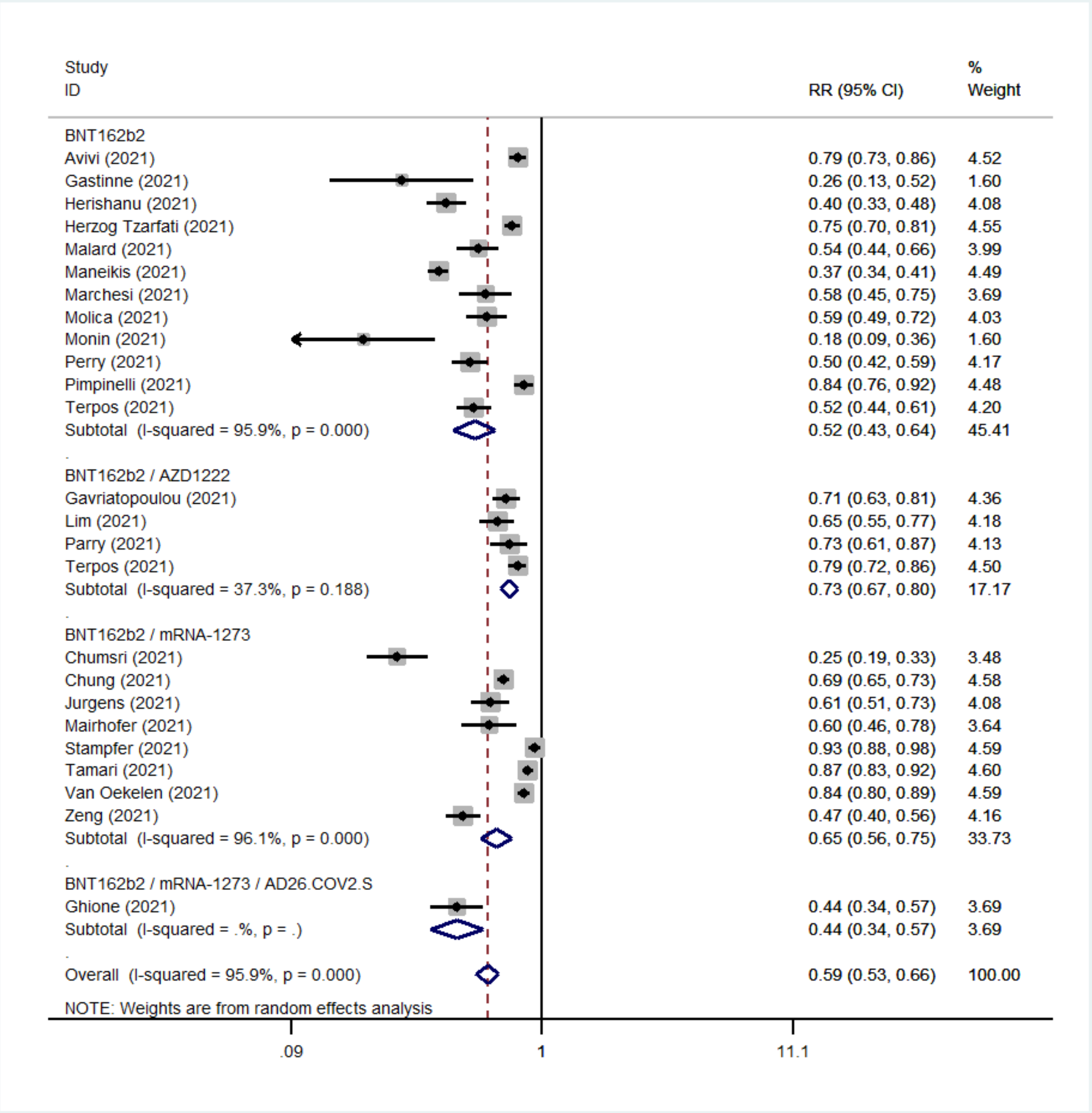

Figure S5. Relative Risk for immune response of patients with hematological malignancies after second vaccine dose.

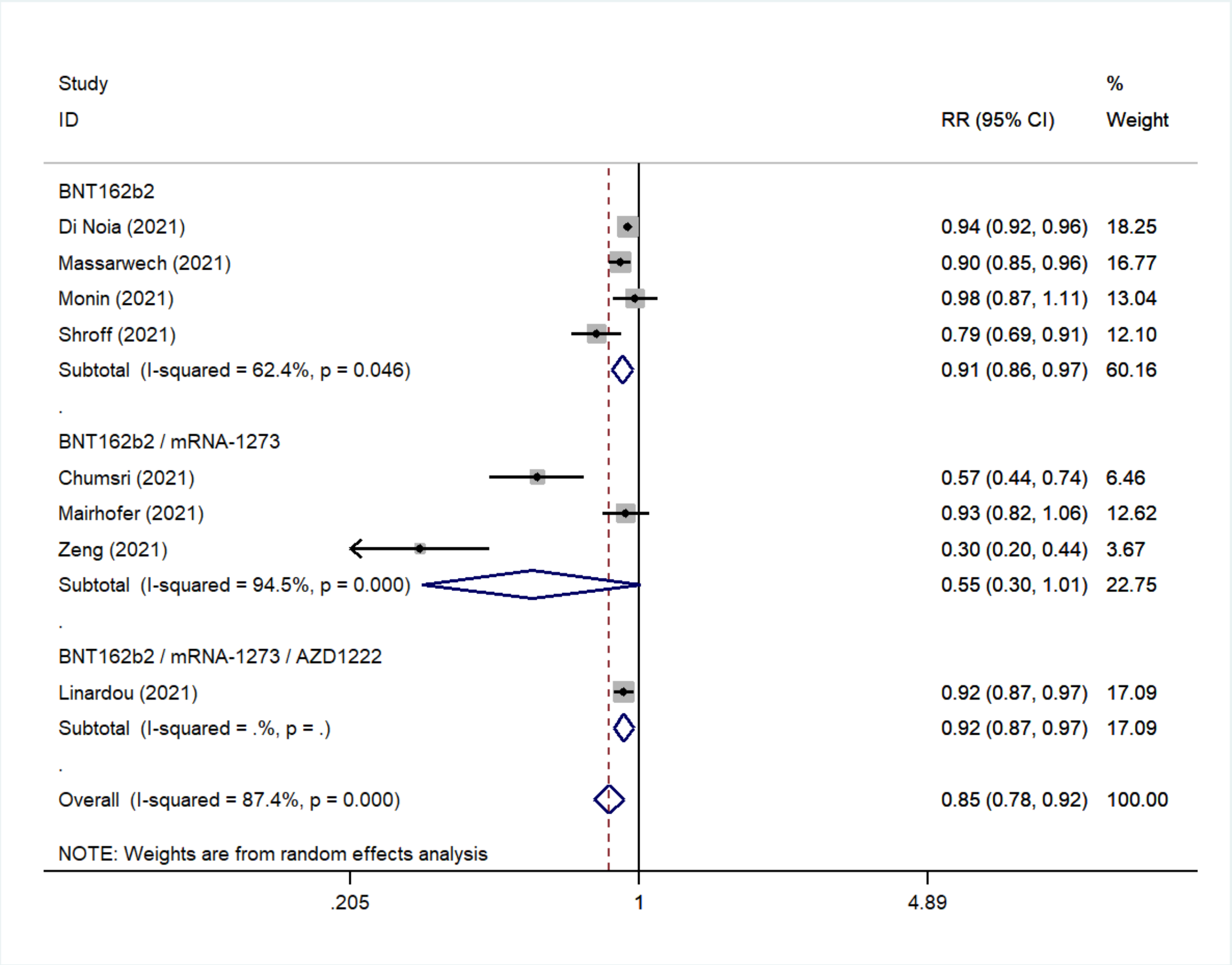

Figure S6. Relative Risk for immune response of patients with solid tumors after second vaccine dose.

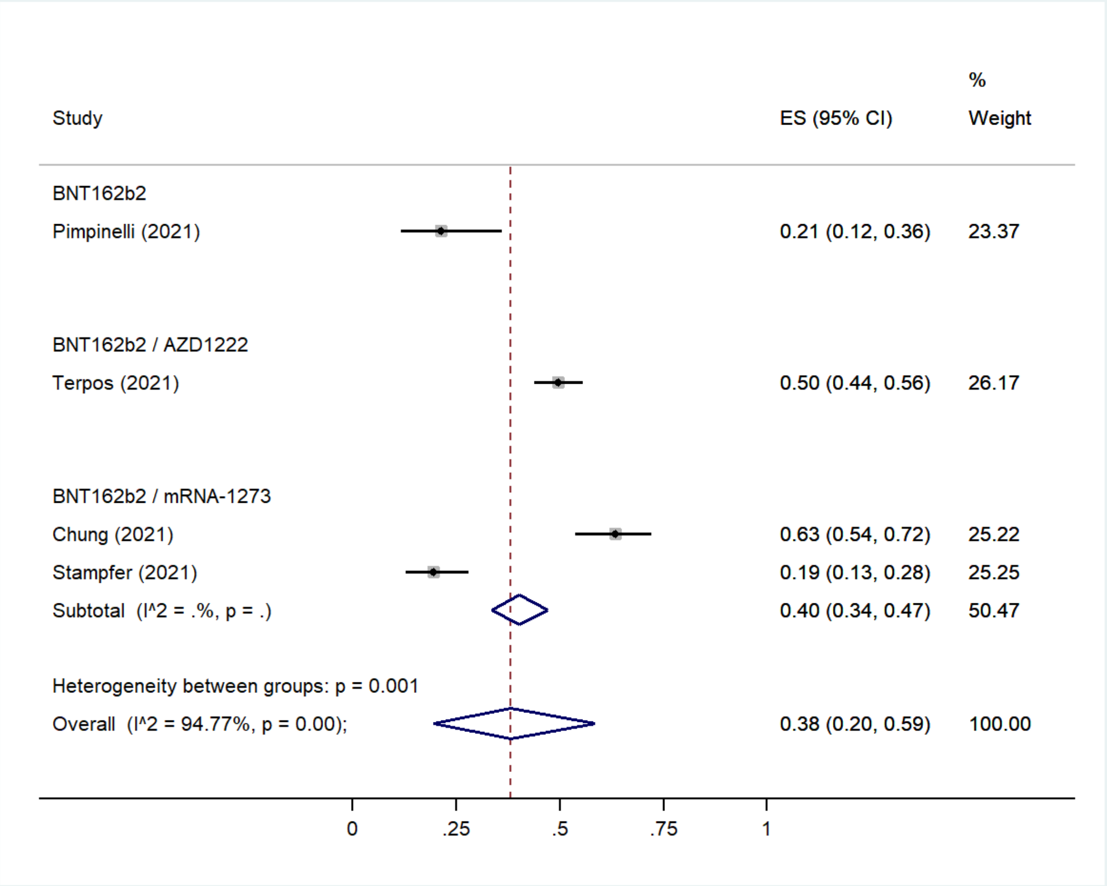

Figure S7. Effect Size for immune seroconversion rates in patients with multiple myeloma after first vaccine dose.

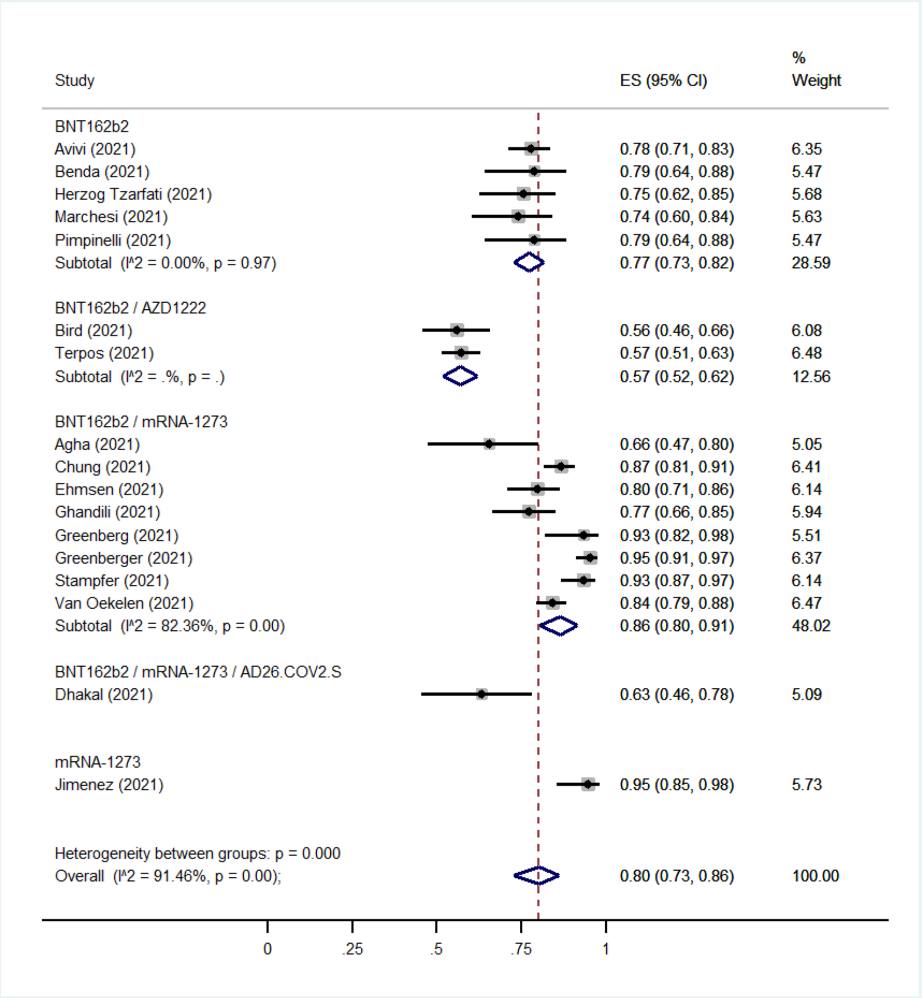

Figure S8. Effect size for immune seroconversion rates of patients with multiple myeloma after second vaccine dose.

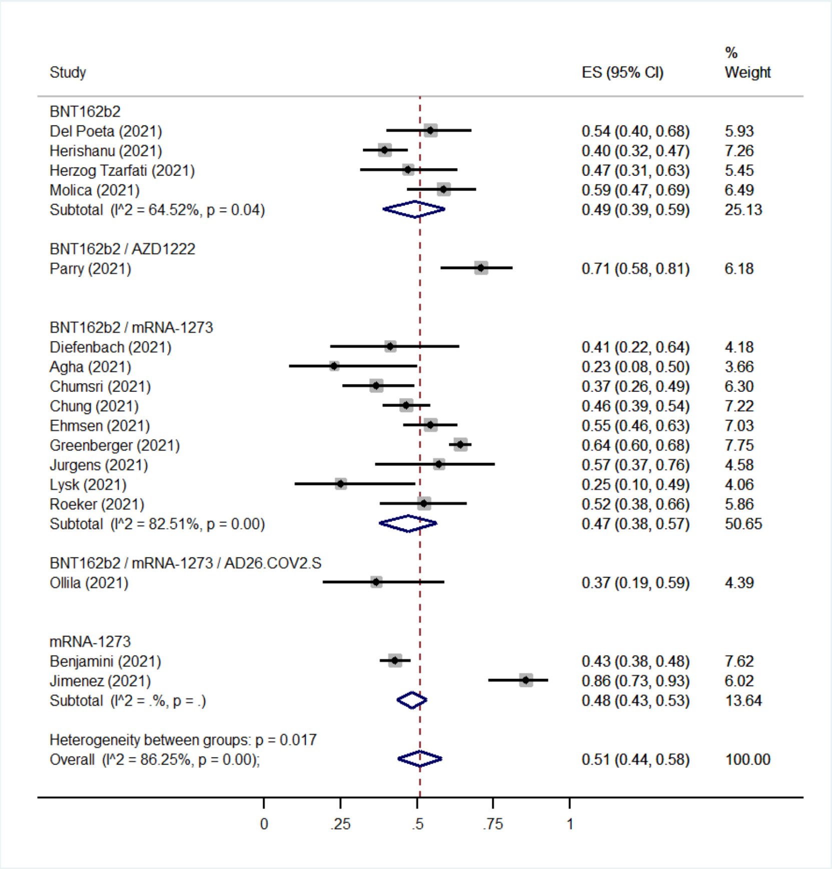

Figure S9. Effect size for immune seroconversion rates of patients with chronic lymphocytic leukemia after second vaccine dose.

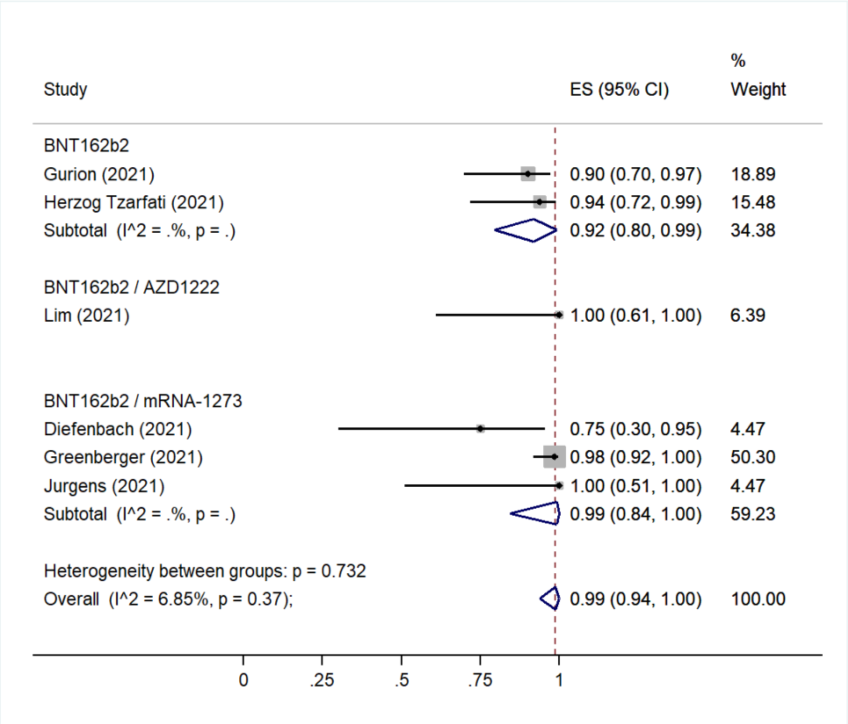

Figure S10. Effect size of immune seroconversion rates of patients with Hodgkin lymphoma after second vaccine dose.

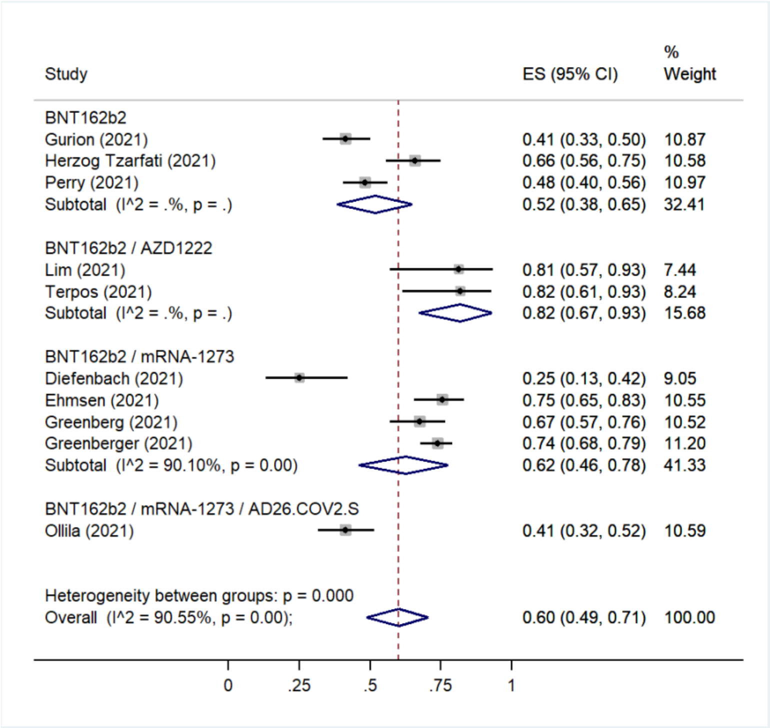

Figure S11. Effect size of immune seroconversion rates of patients with non-Hodgkin myeloma after second vaccine dose.

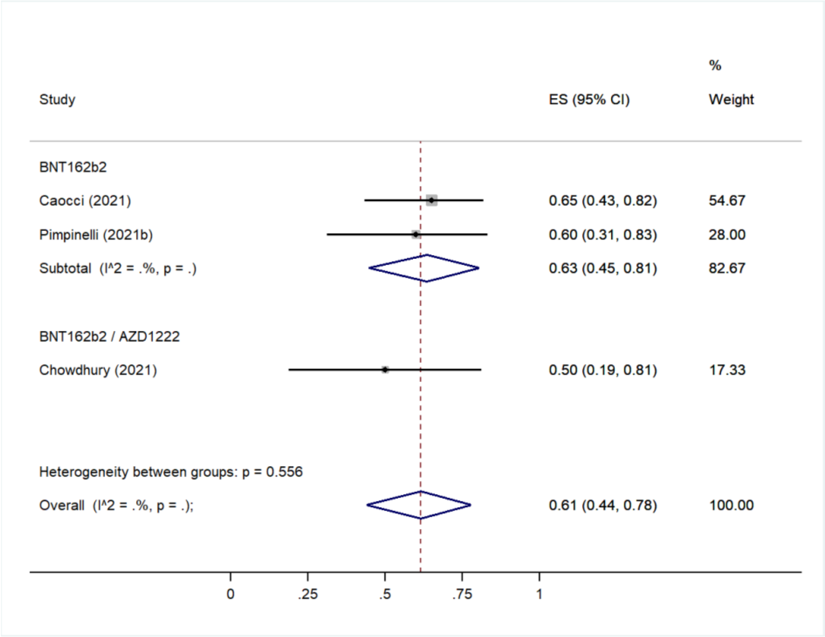

Figure S12. Effect size of immune seroconversion rates of patients with myelofibrosis after second vaccine dose.

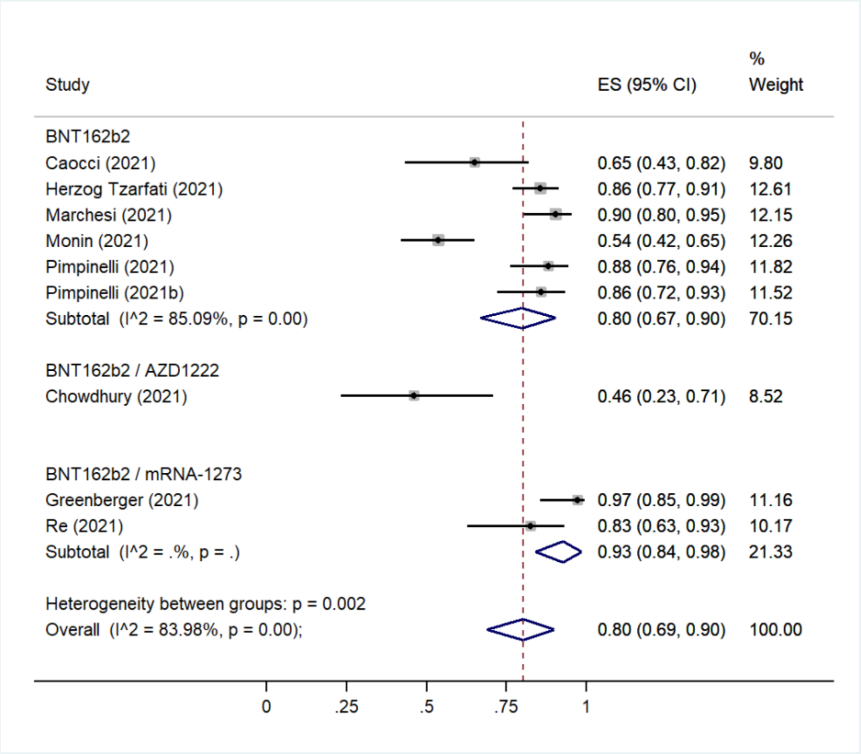

Figure S13. Effect Size for immune seroconversion rates of patients with myelodysplastic and myeloproliferative syndromes after second vaccine dose.

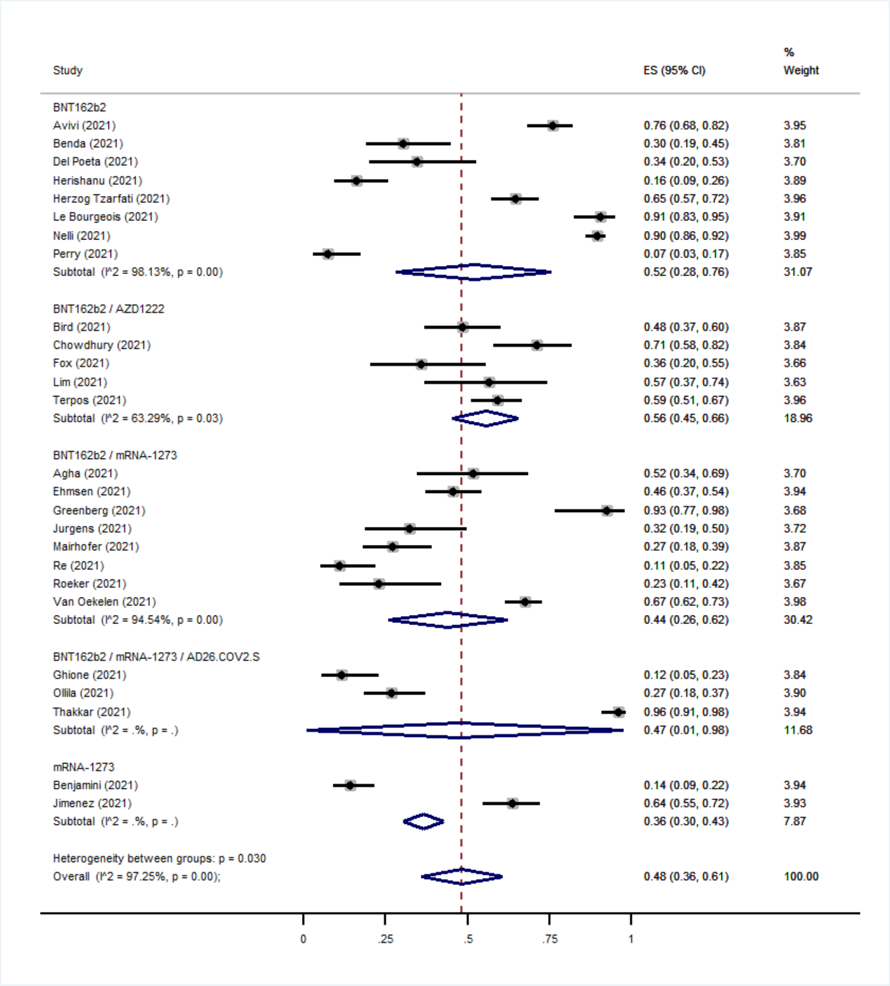

Figure S14. Effect Size for immune seroconversion rates of patients under active treatment after second vaccine dose.

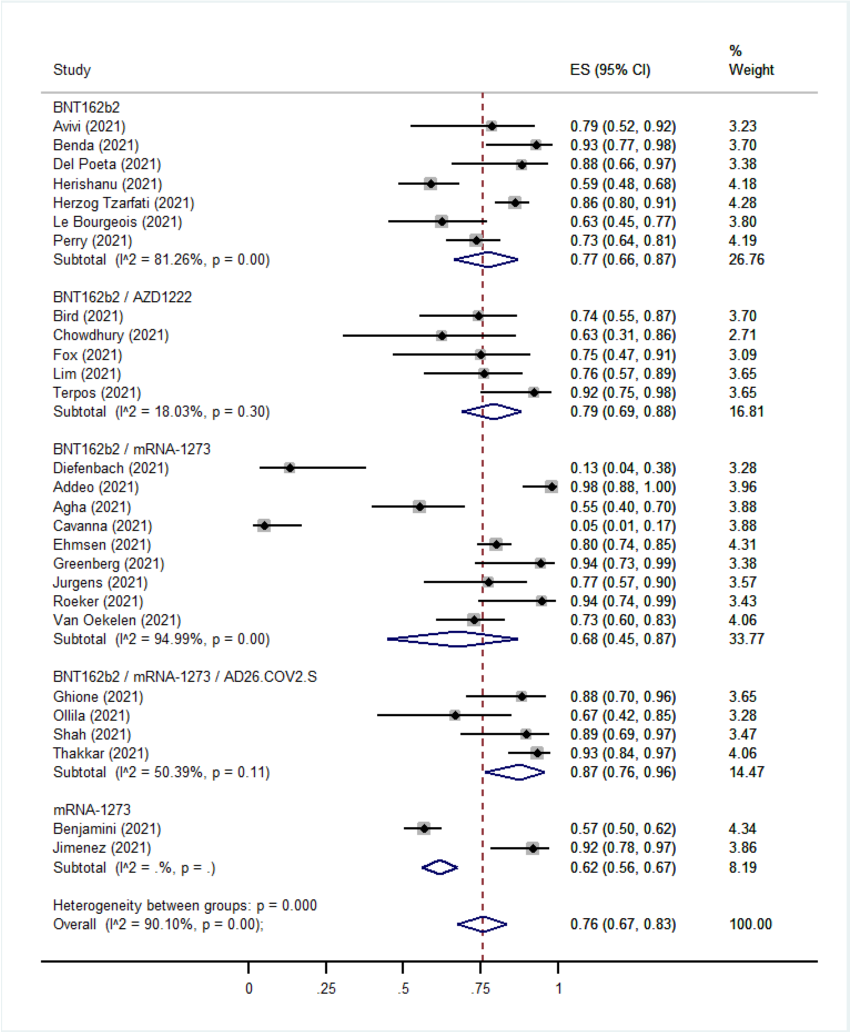

Figure S15. Effect Size for immune seroconversion rates of treatment naïve patients after second dose.

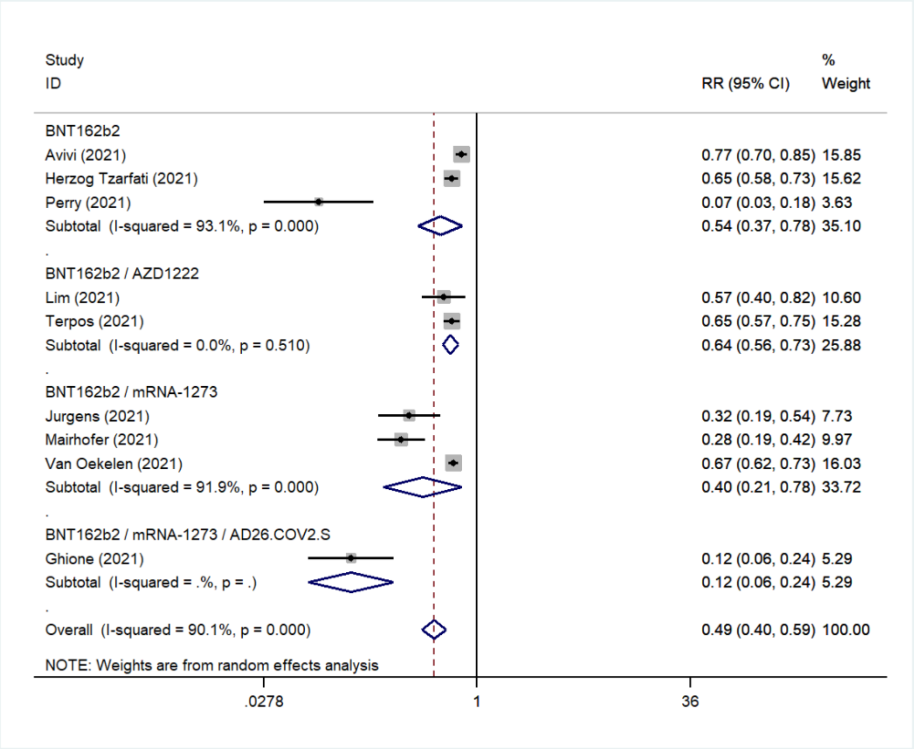

Figure S16. Relative Risk for immune seroconversion rates of patients under active treatment after second vaccine dose.

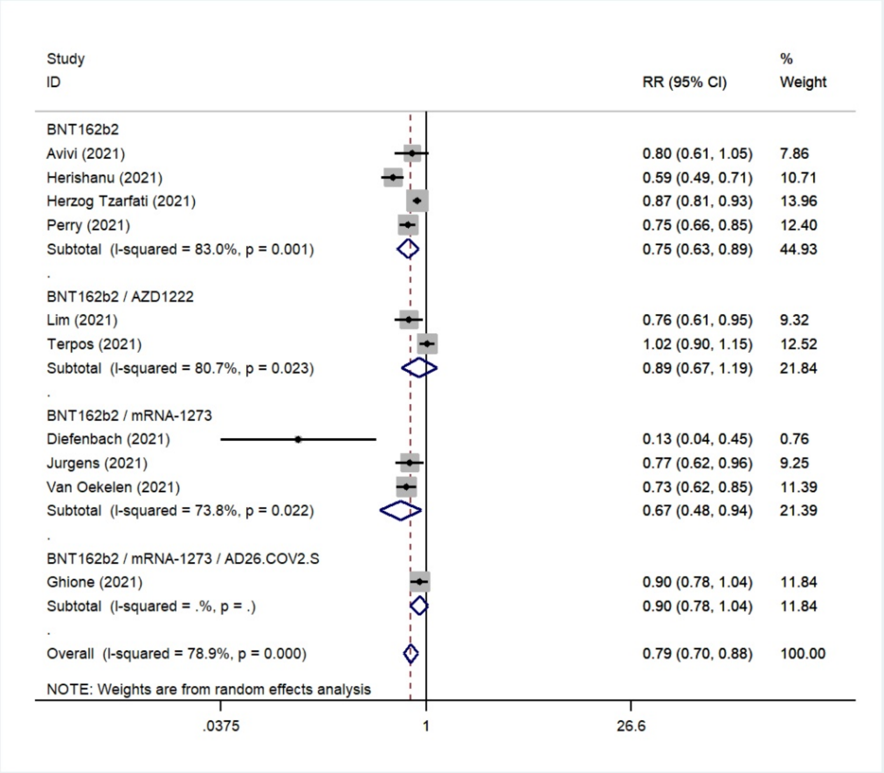

Figure S17. Relative Risk for immune seroconversion rates of treatment naïve patients after second vaccine dose.

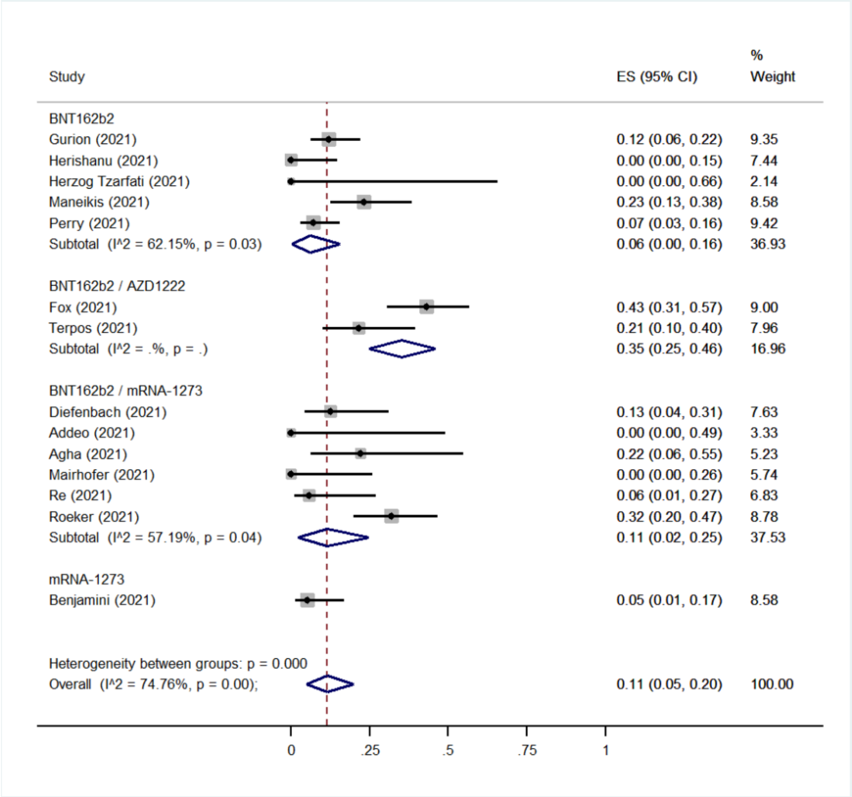

Figure S18. Effect Size for immune seroconversion rates of patients under active anti-CD20 monoclonal antibodies after second vaccine dose.

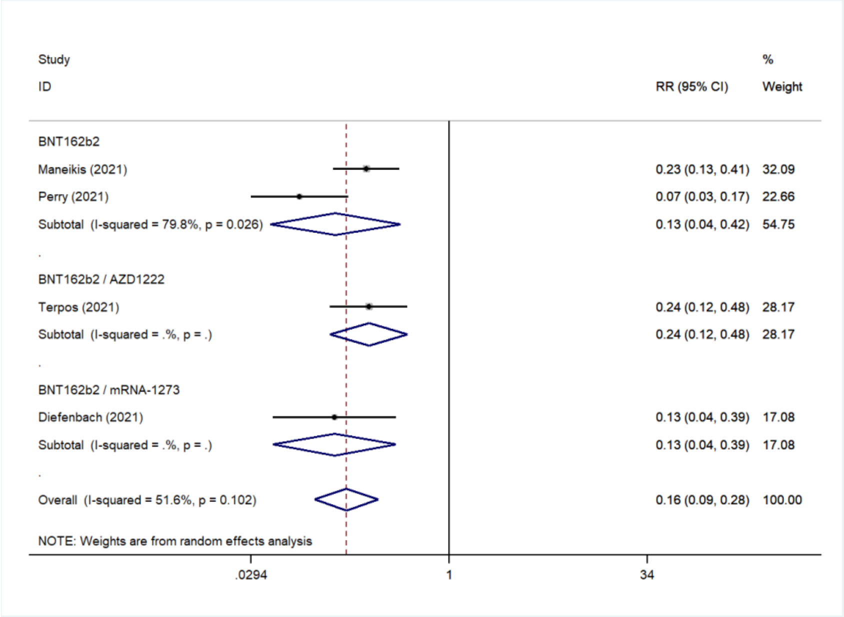

Figure S19. Relative Risk of immune seroconversion rates of patients under active anti-CD20 monoclonal antibodies after second vaccine dose.

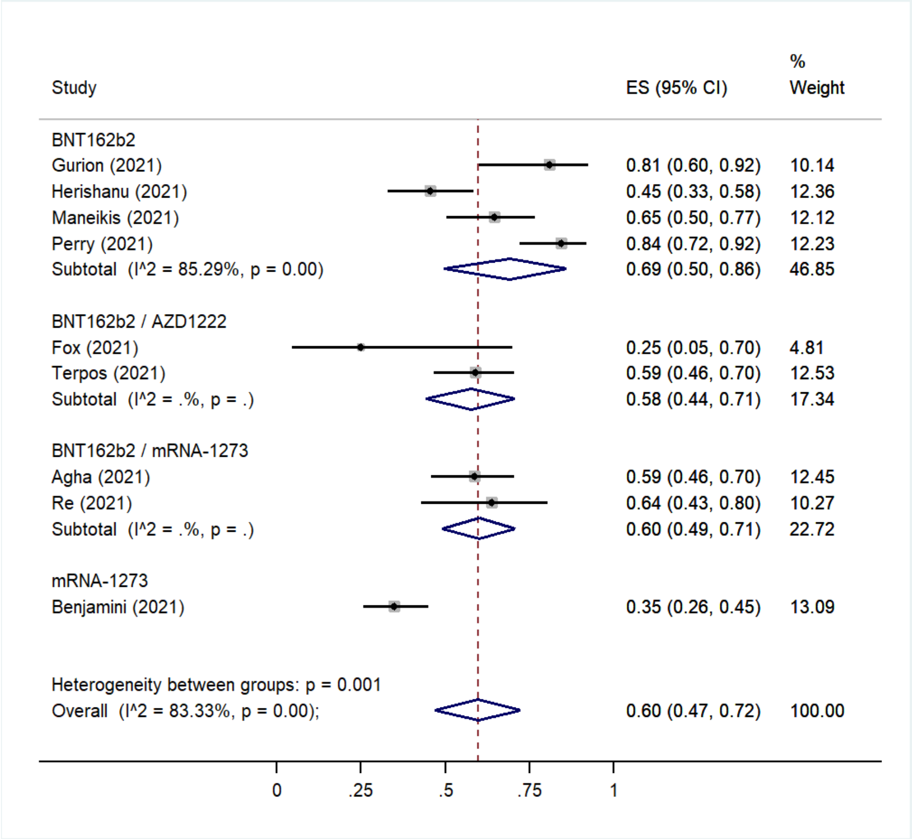

Figure S20. Effect Size for immune seroconversion rates of patients having received treatment with anti-CD20 longer than one year ago.

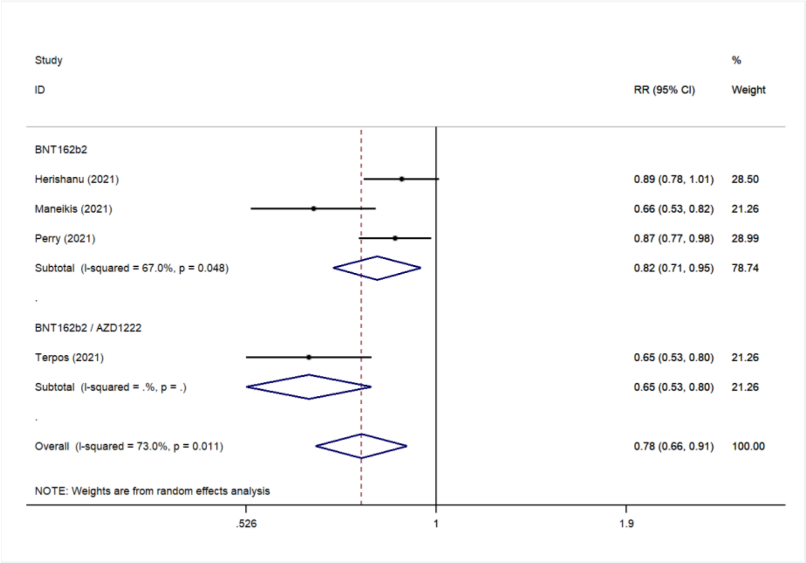

Figure S21. Relative Risk for immune seroconversion rates of patients having received treatment with anti-CD20 longer than one year ago.

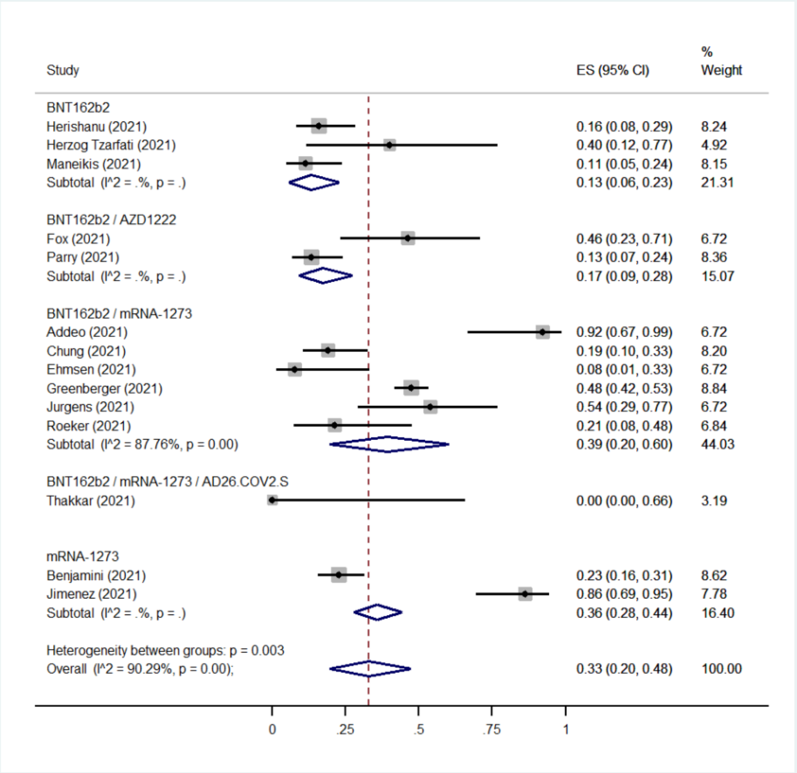

Figure S22. Effect Size of immune seroconversion rates of patients under active Bruton's Tyrosine Kinase inhibitors treatment after second dose.

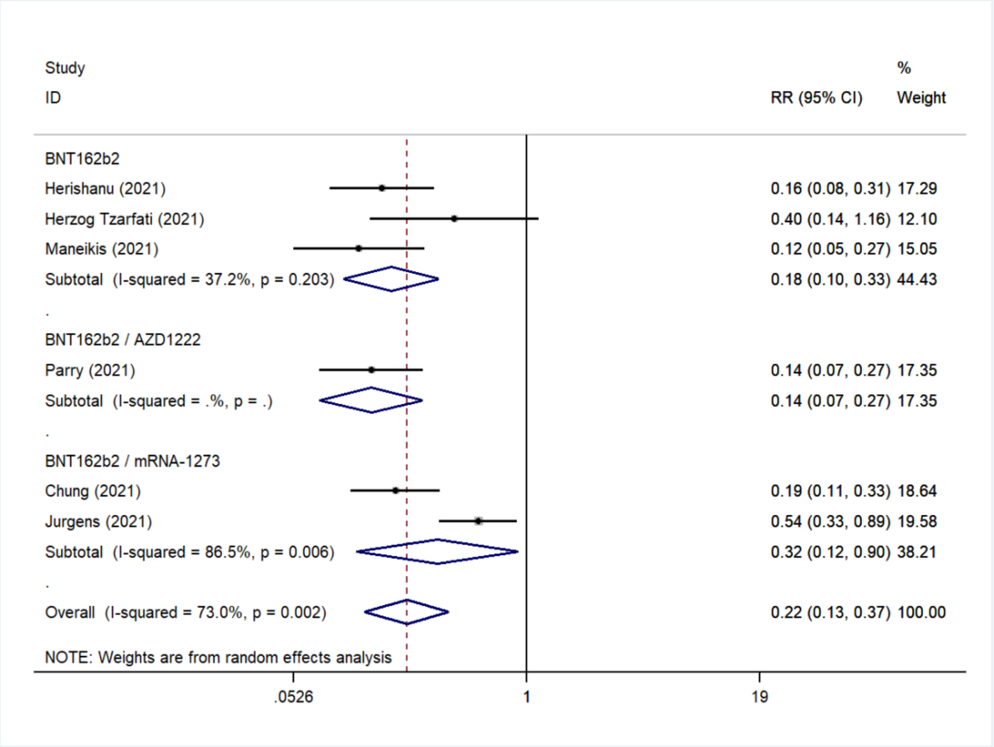

Figure S23. Relative Risk of immune seroconversion rates of patients under active Bruton's Tyrosine kinase inhibitors treatment after second dose.

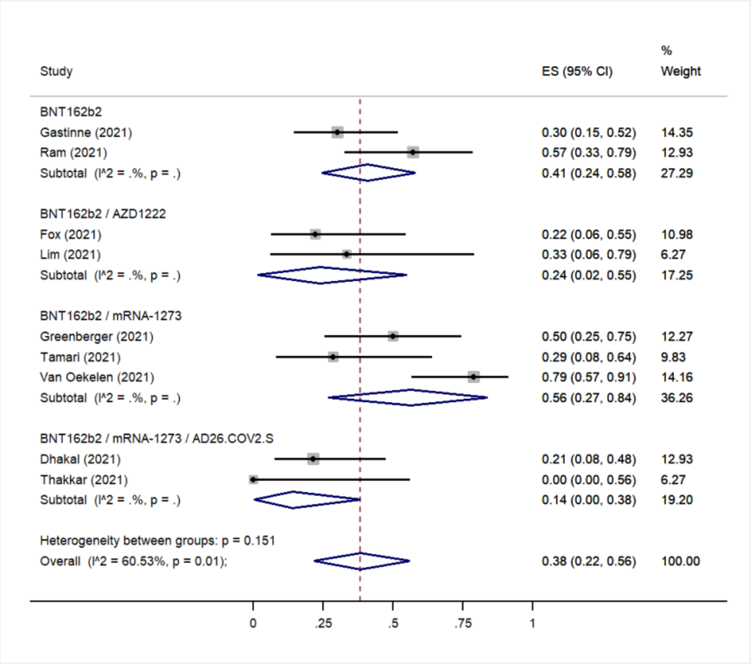

Figure S24. Effect Size for immune seroconversion rates of patients under CAR-T cells therapy after second vaccine dose.

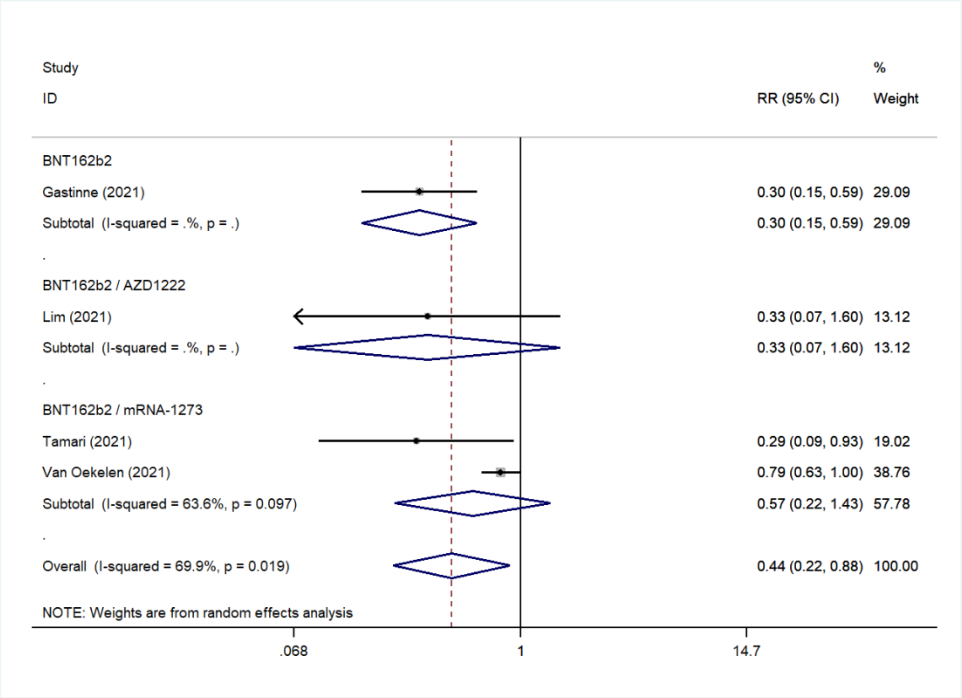

Figure S25. Relative Risk of immune seroconversion rate of patients under CAR-T cells therapy after second vaccine dose.

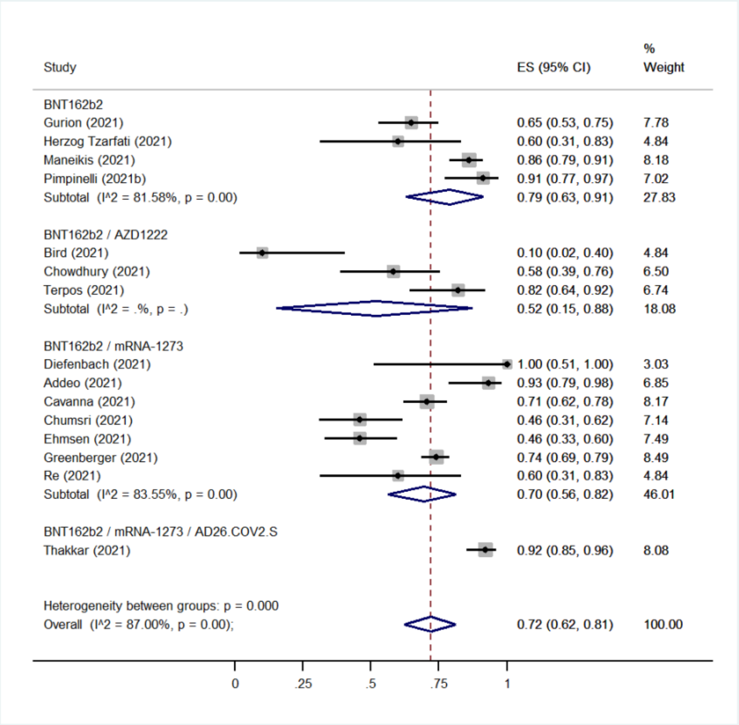

Figure S26. Effect Size for immune seroconversion rates of patients under chemotherapy after second vaccine dose.

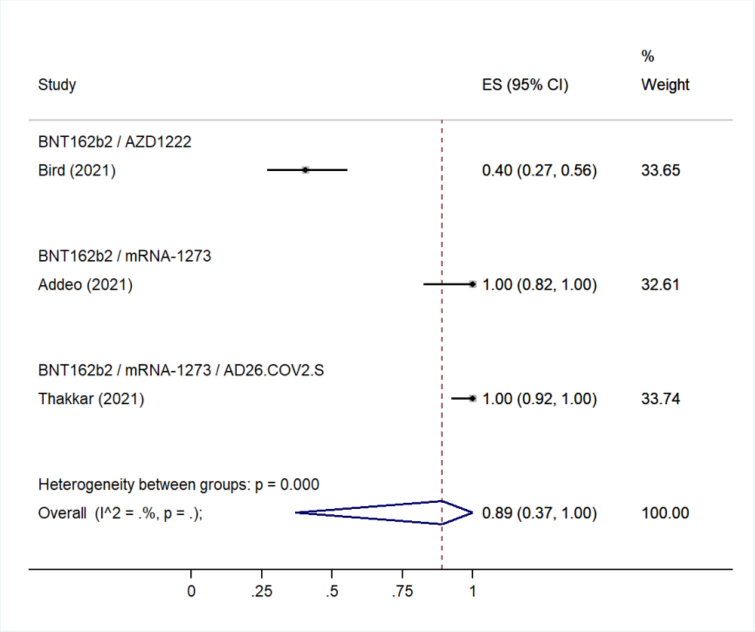

Figure S27. Effect Size for immune seroconversion rates of patients under endocrine therapy after second vaccine dose.

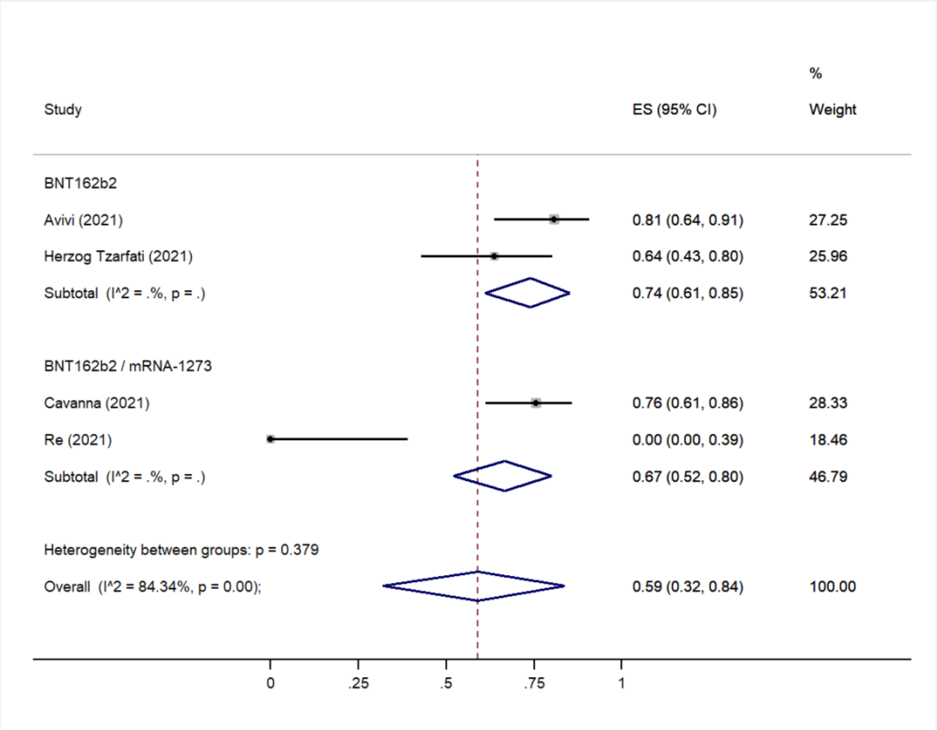

Figure S28. Effect Size for immune seroconversion rates of patients under combination treatment after second vaccine dose.

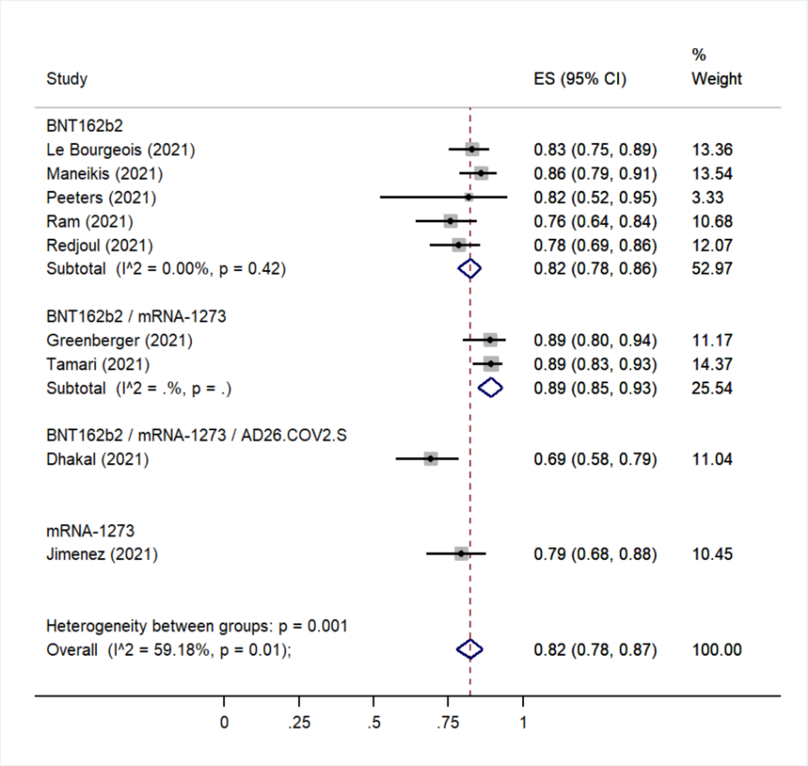

Figure S29. Effect size for immune seroconversion rates of patients who underwent allogeneic stem cell transplantation after second vaccine dose.

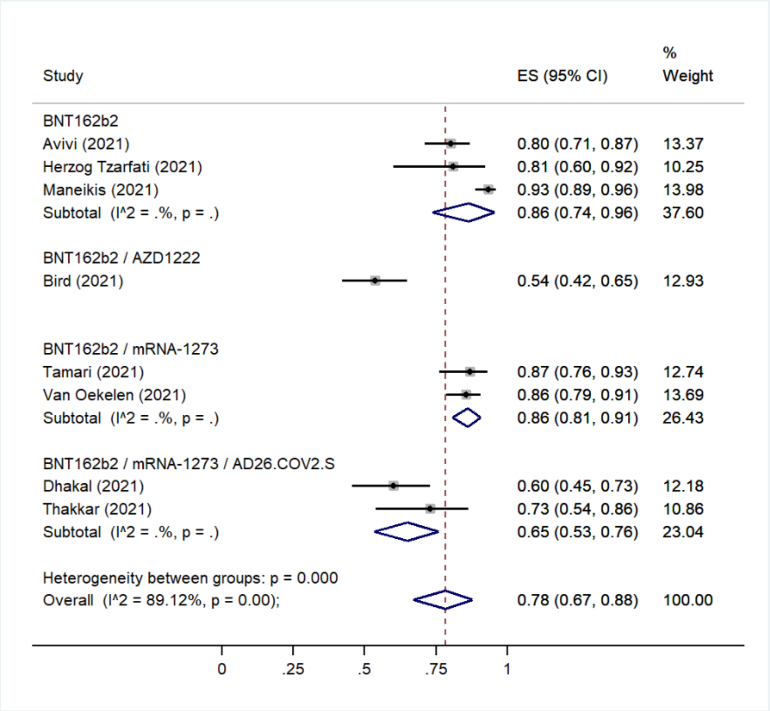

Figure S30. Effect Size for immune seroconversion rates of patients who underwent autologous stem cell transplantation after second vaccine dose.

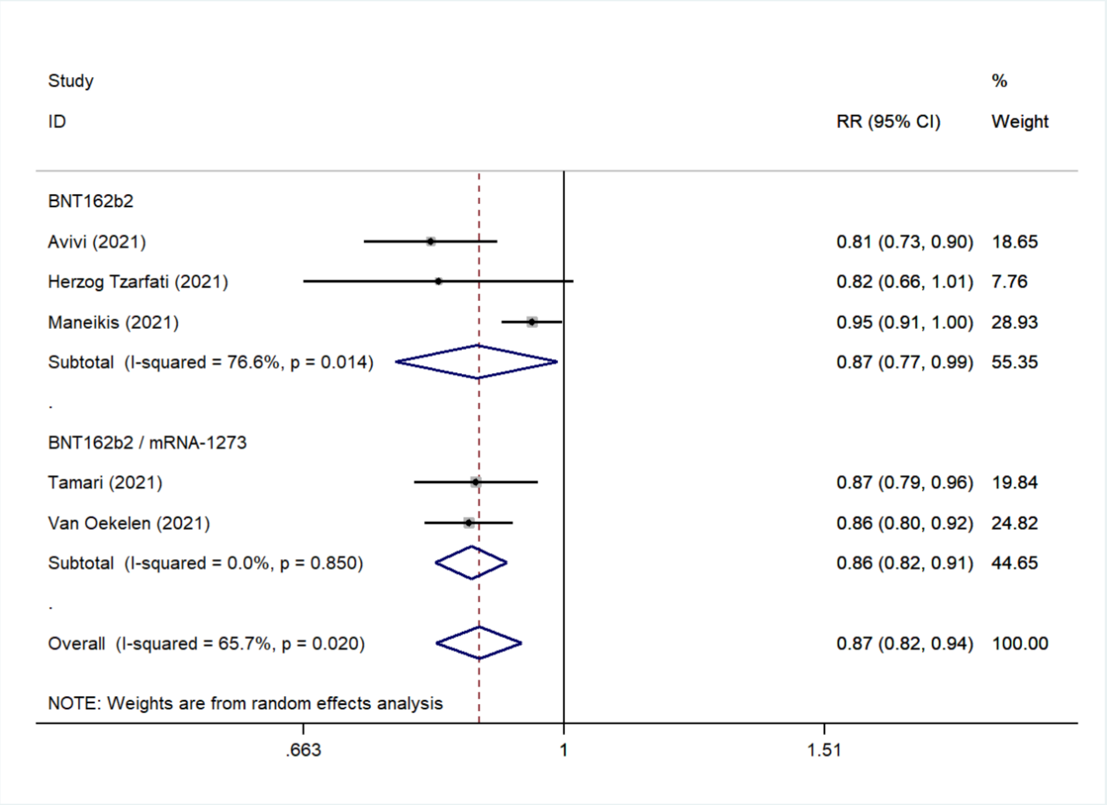

Figure S31. Relative Risk for immune seroconversion rates of patients who underwent allogeneic stem cell transplantation after second vaccine dose.

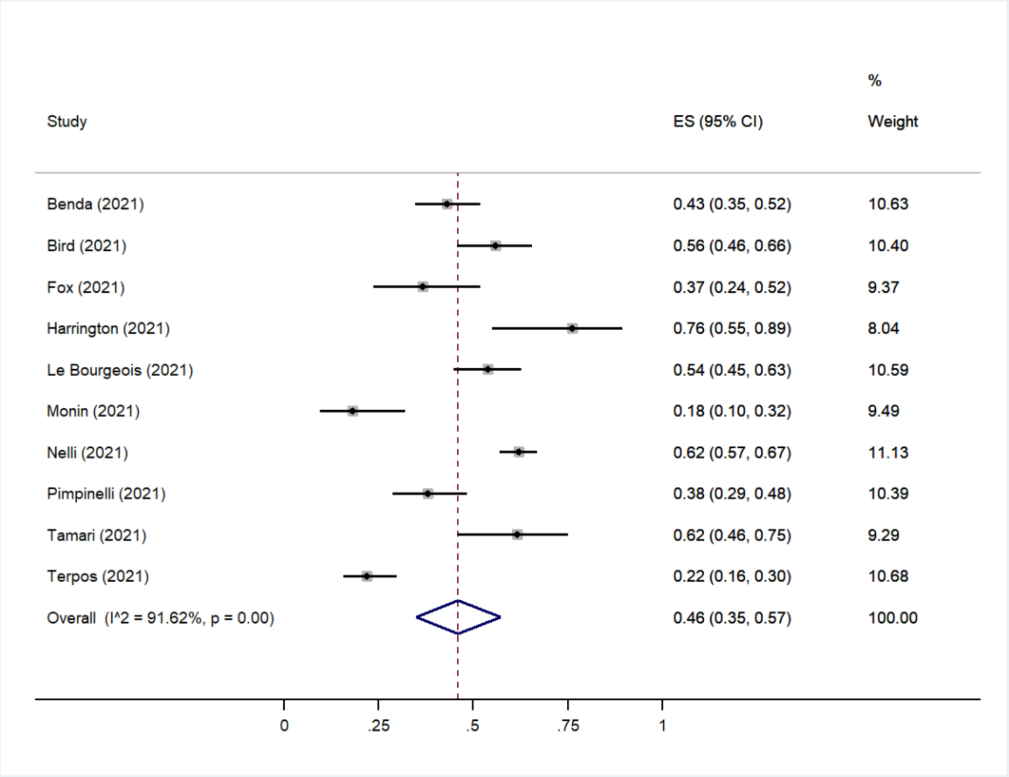

Figure S32. Effect Size for immune seroconversion rates in patients with hematological malignancies who received first dose of BNT162B2 vaccine.

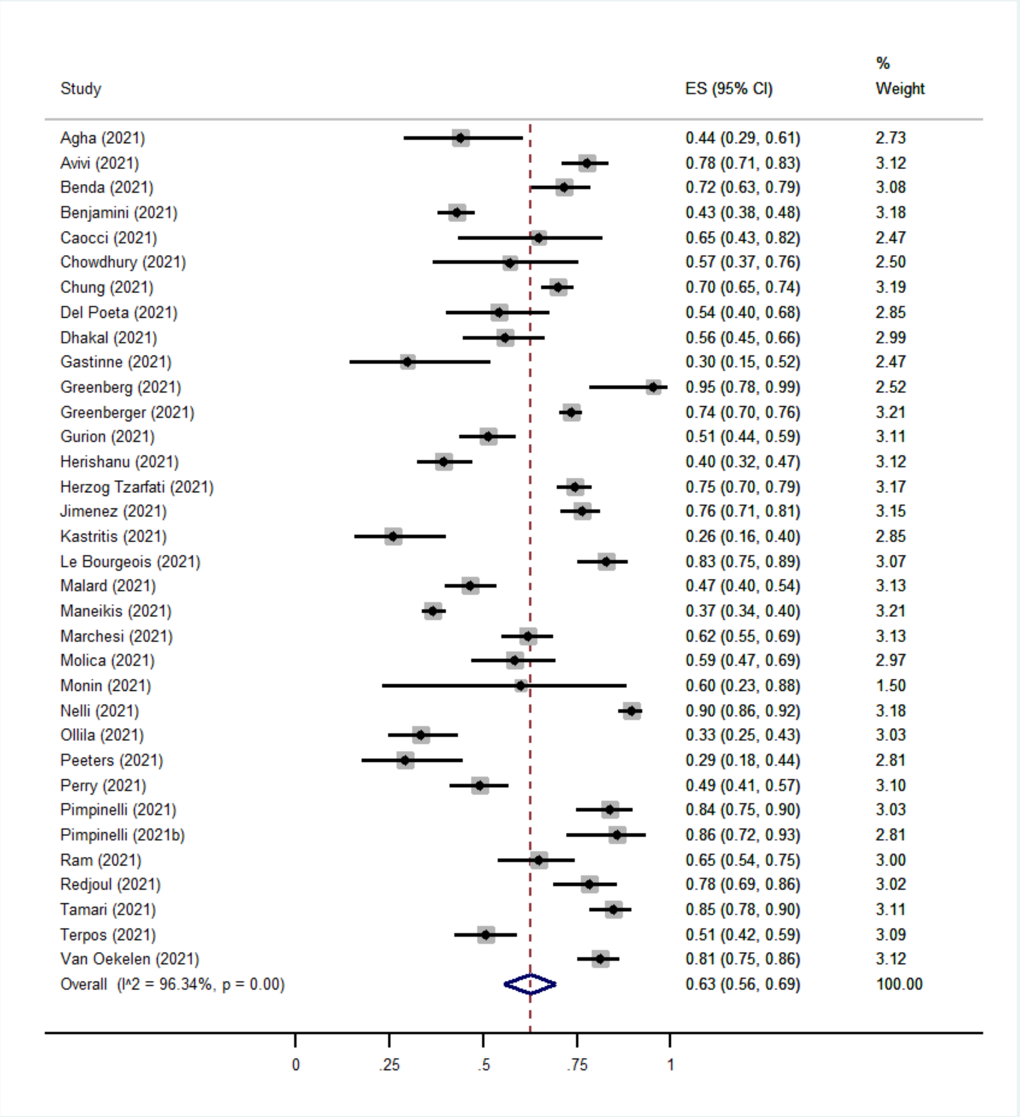

Figure S33. Effect Size of immune seroconversion rates in patients with hematological malignancies who received second dose of BNT162B2.

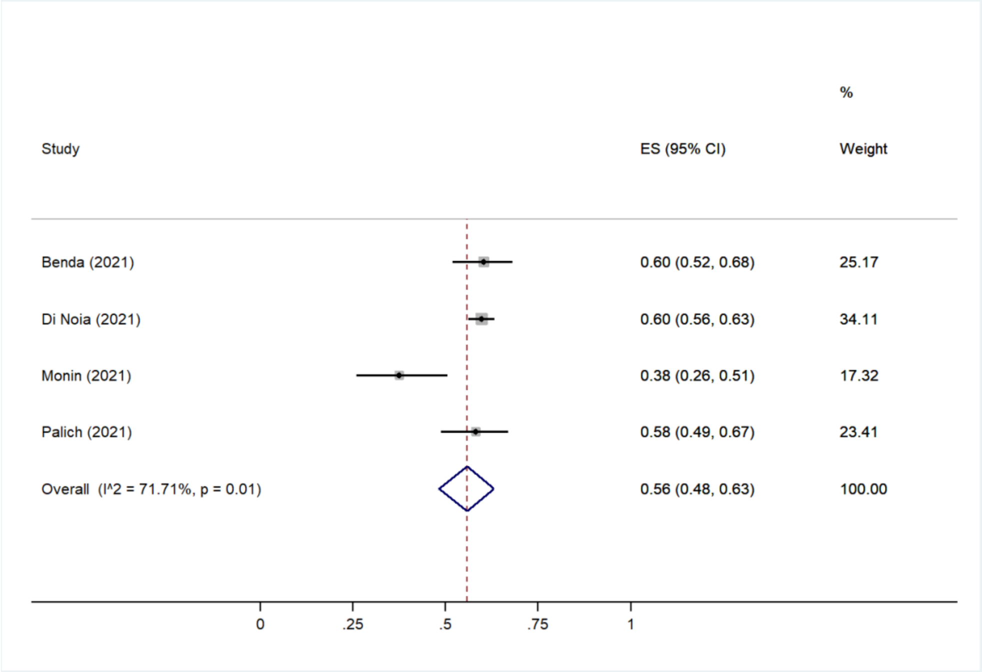

Figure S34. Effect Size for immune seroconversion rates of patients with solid tumors who received first dose of BNT162B2 vaccine.

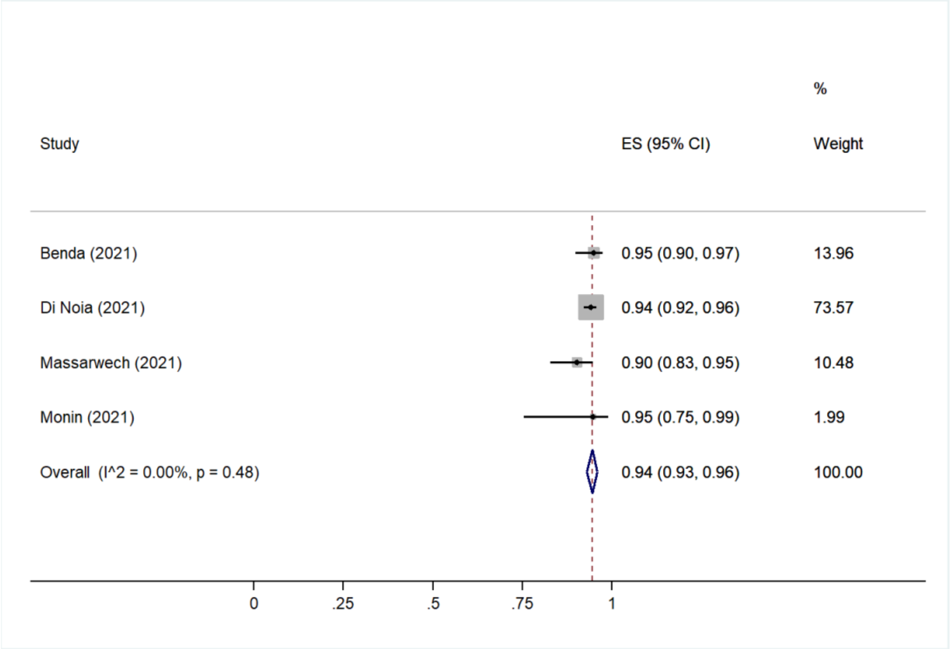

Figure S35. Effect Size for immune seroconversion rates of patients with solid tumors who received second dose of BNT162B2 vaccine.

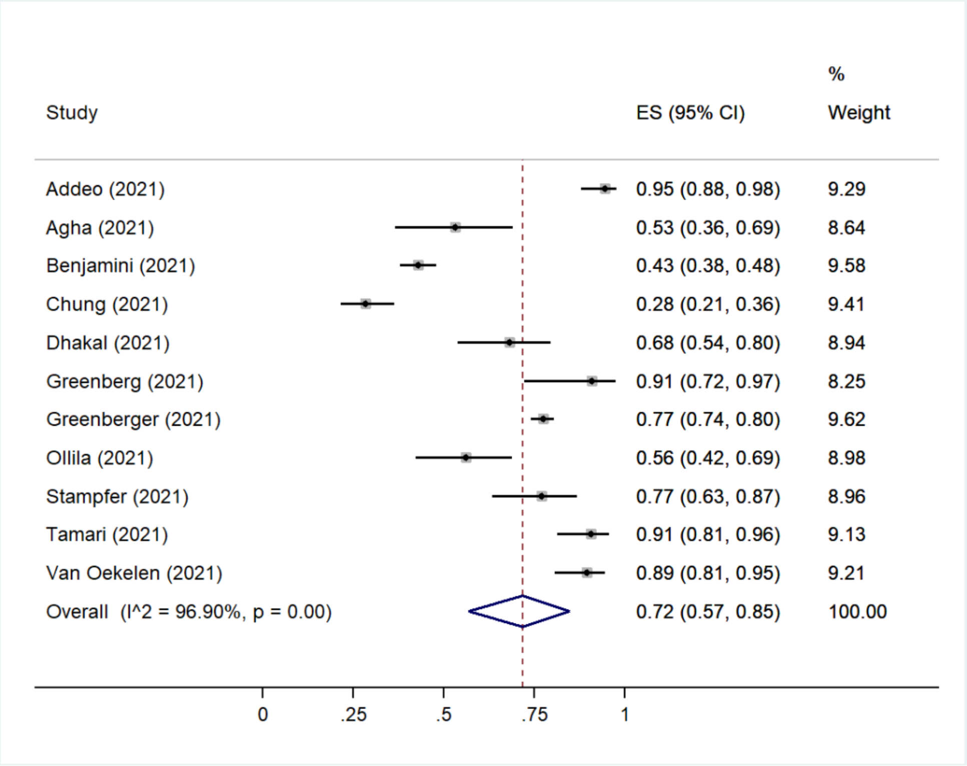

Figure S36. Effect Size for immune seroconversion rates of patients with hematological malignancies after second mRNA-1273.

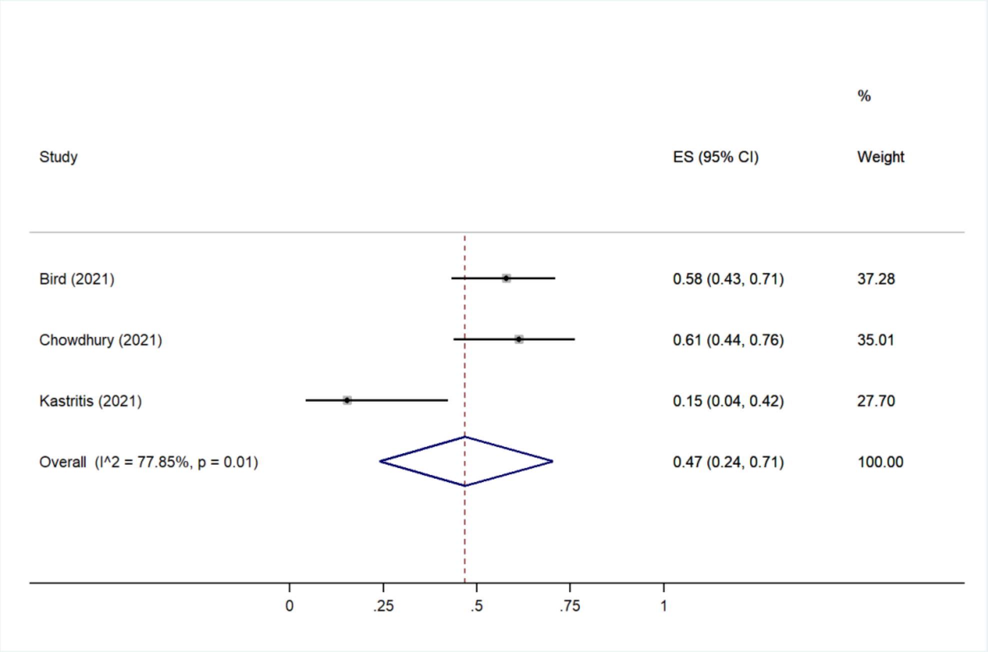

Figure S37. Effect Size for immune seroconversion rates of patients with hematological malignancies after second AZD1222.

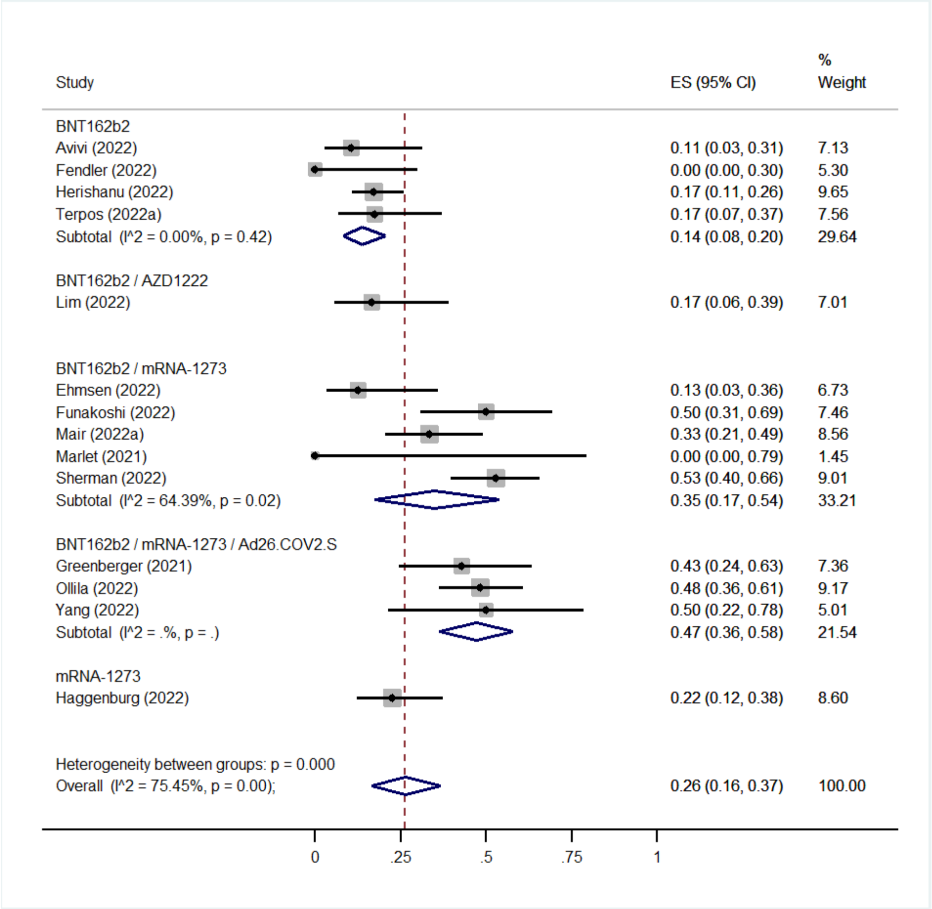

Figure S38. Effect Size for immune seroconversion rates of patients on active therapy with anti-CD20 monoclonal antibodies after third vaccine dose.

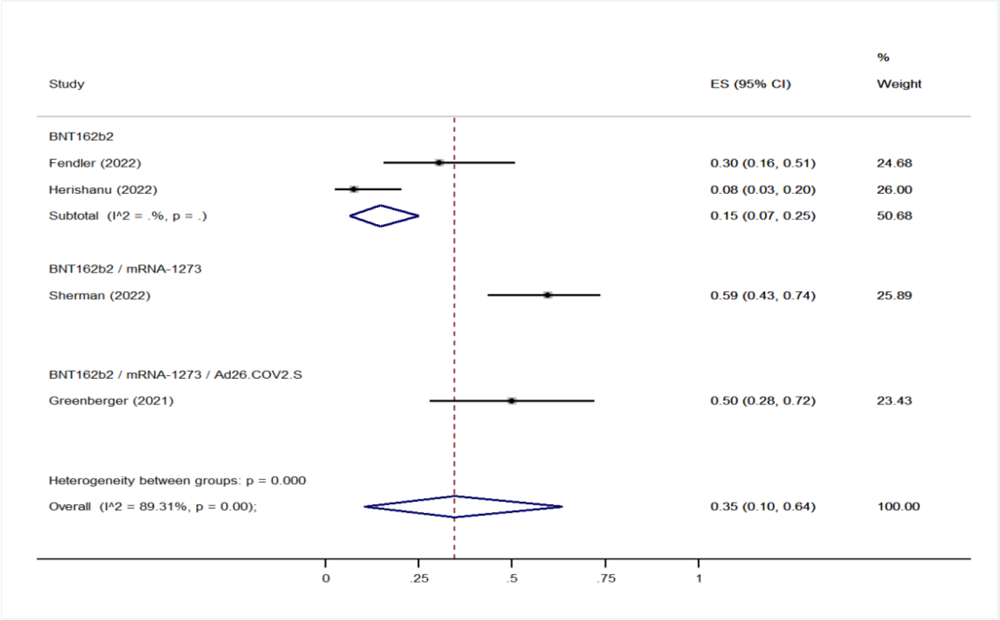

Figure S39. Effect Size for immune seroconversion rates of patients on active therapy with combination therapy after third vaccine dose.

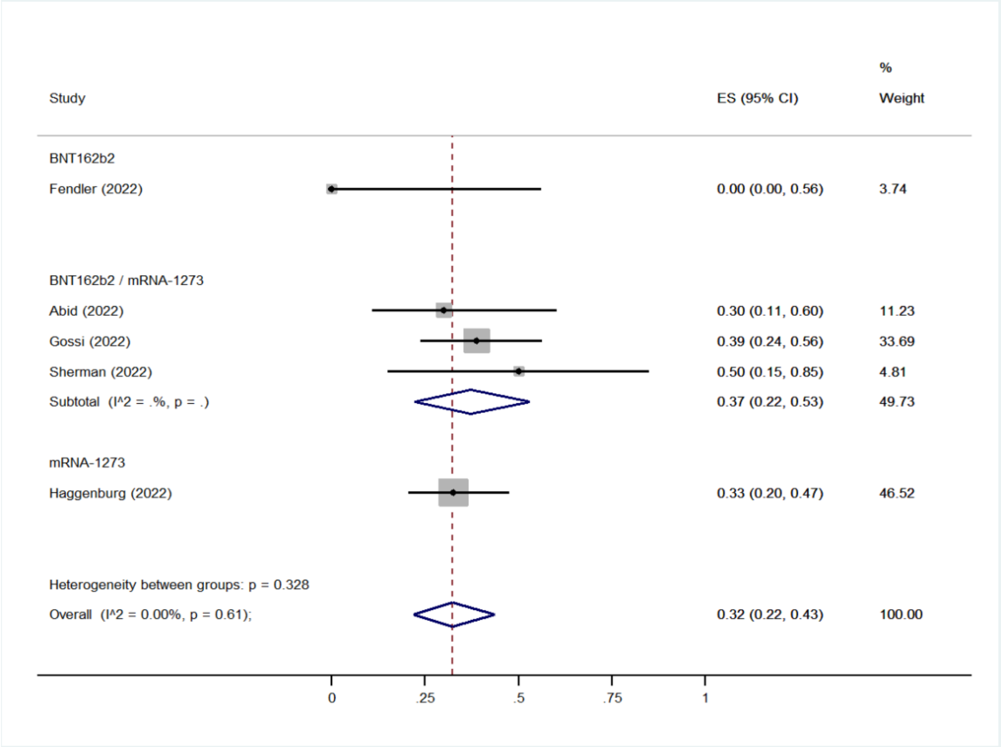

Figure S40. Effect Size for immune seroconversion rates of patients under active CAR-T cell therapy after third vaccine dose.

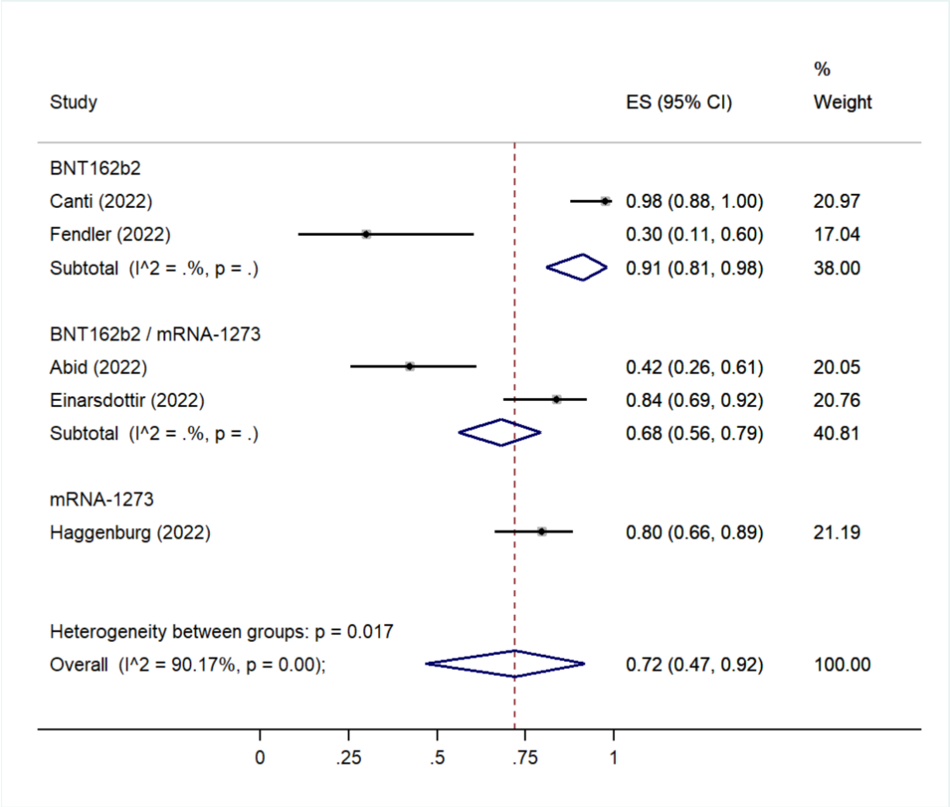

Figure S41. Effect Size for immune seroconversion rates of patients who underwent allogenic stem cell transplant after third vaccine dose.

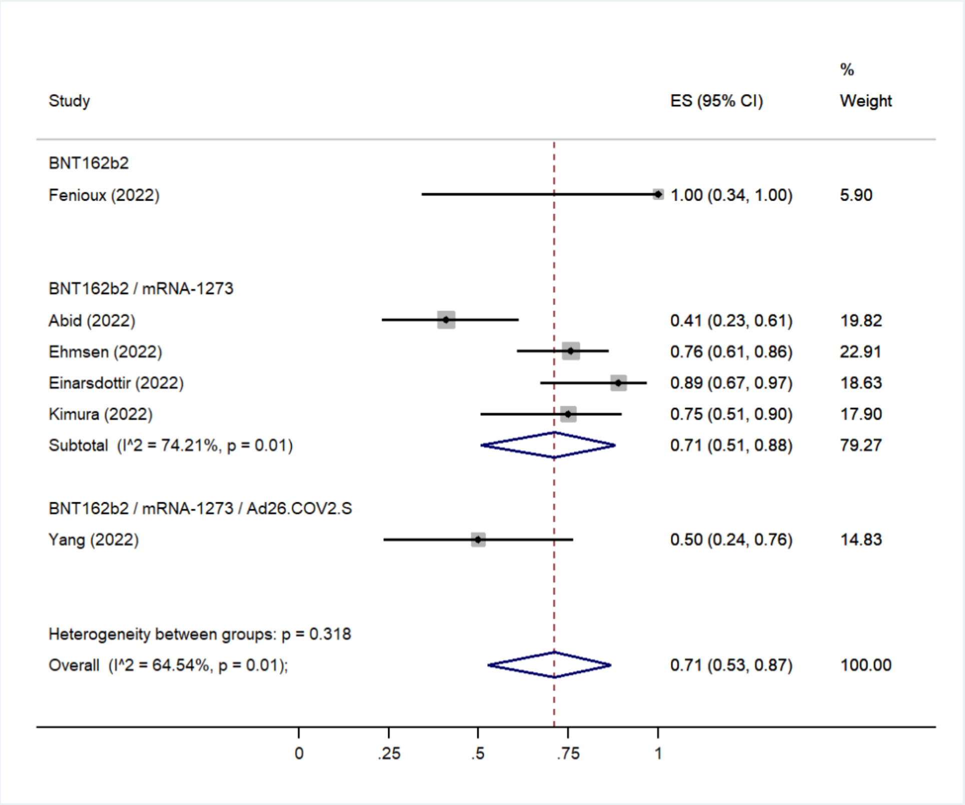

Figure S42. Effect Size for immune seroconversion rates of patients on treatment with steroids after third vaccine dose.

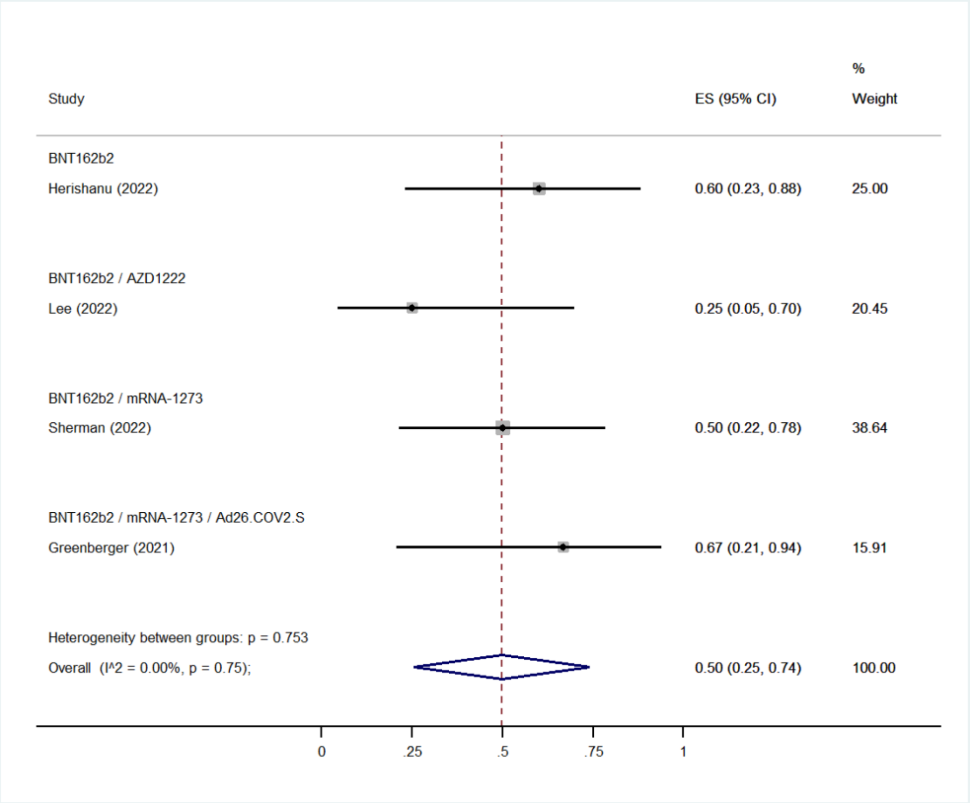

Figure S43. Effect Size for immune seroconversion rates of patients under venetoclax therapy after third vaccine dose.

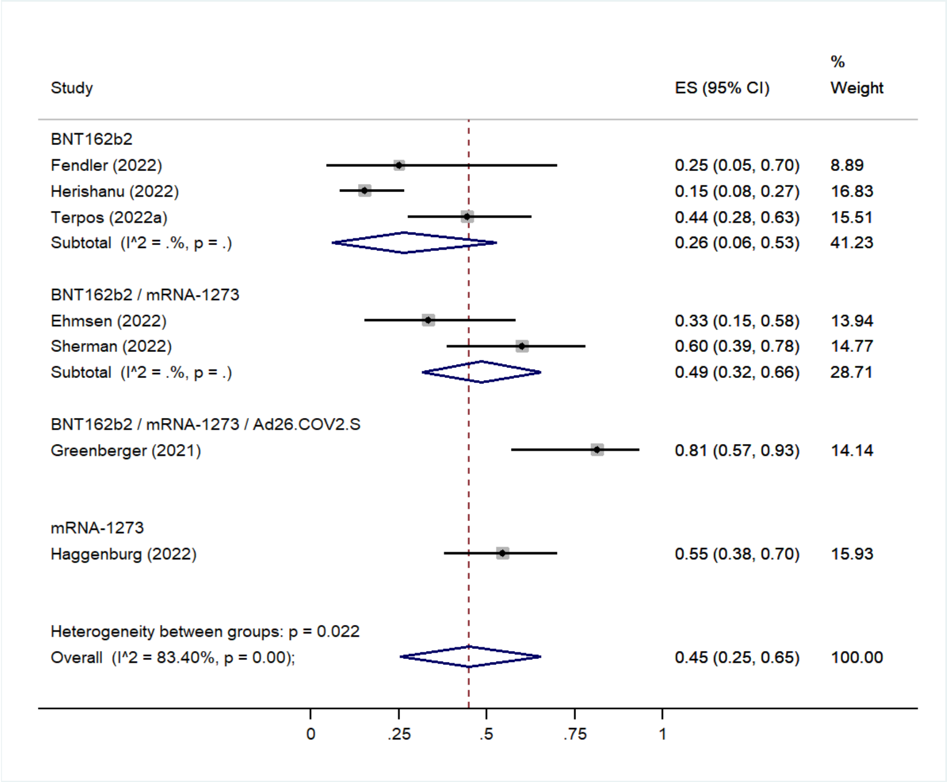

Figure S44. Effect Size for immune seroconversion rates of patients on Bruton's Tyrosine Kinase inhibitors after third vaccine dose.

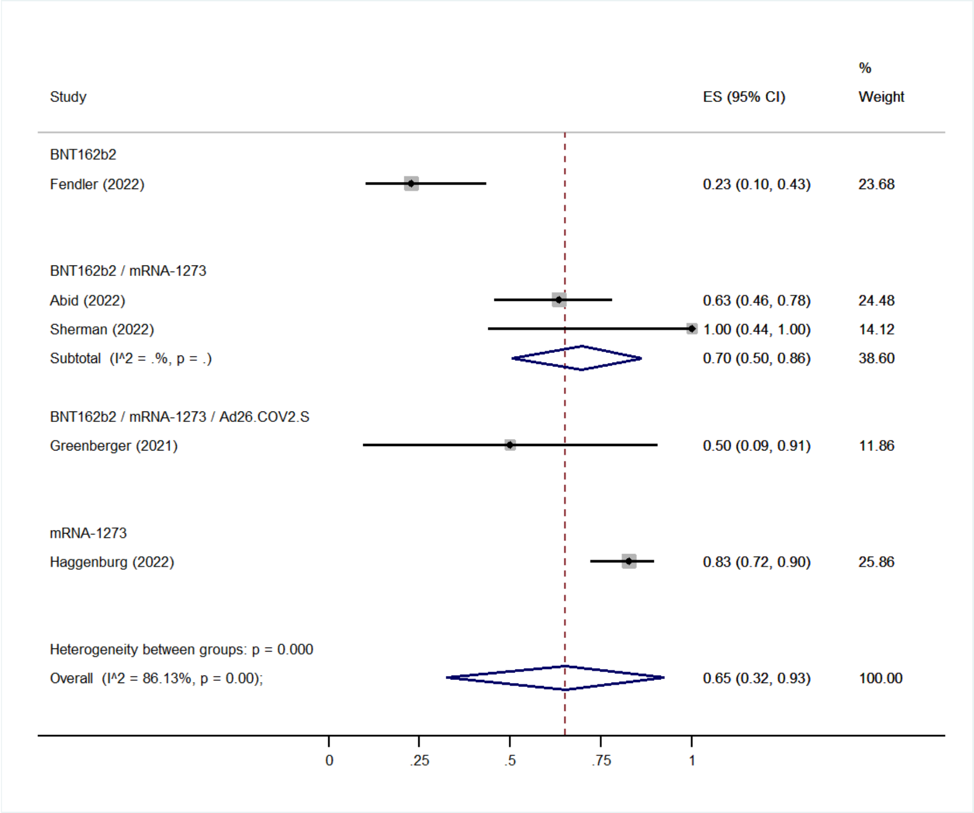

Figure S45. Effect size for immune seroconversion rates of patients who underwent autologous stem cell transplantation after third vaccine dose.

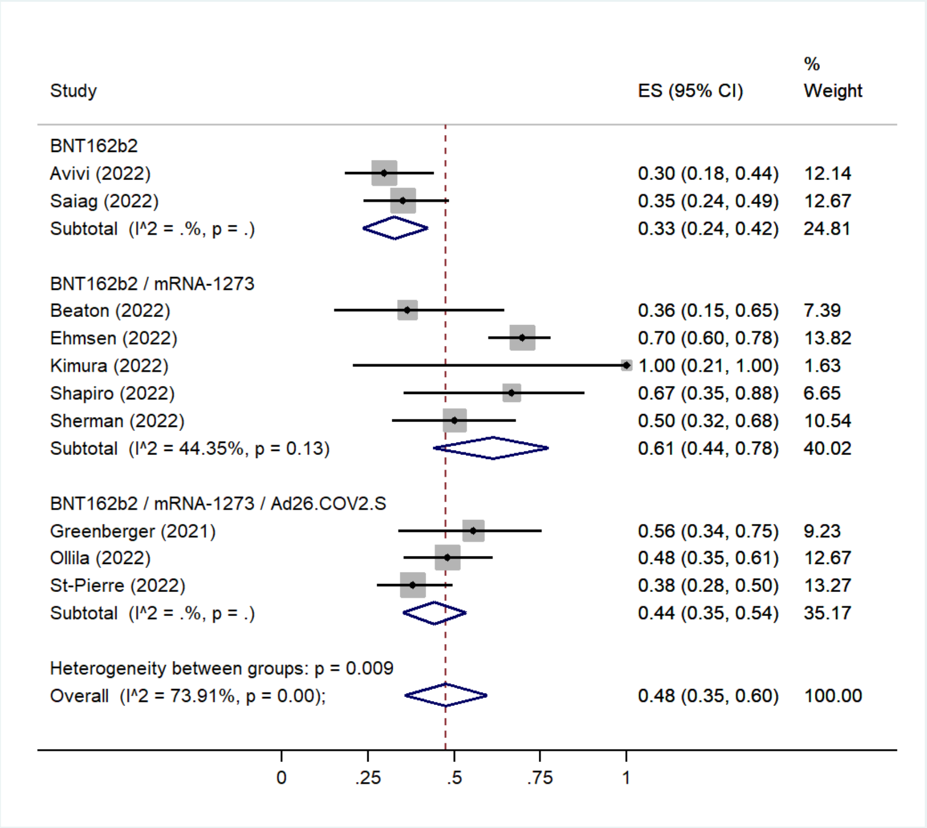

Figure S46. Effect Size for immune seroconversion rates of patients with non-Hodgkin lymphoma after third vaccine dose.

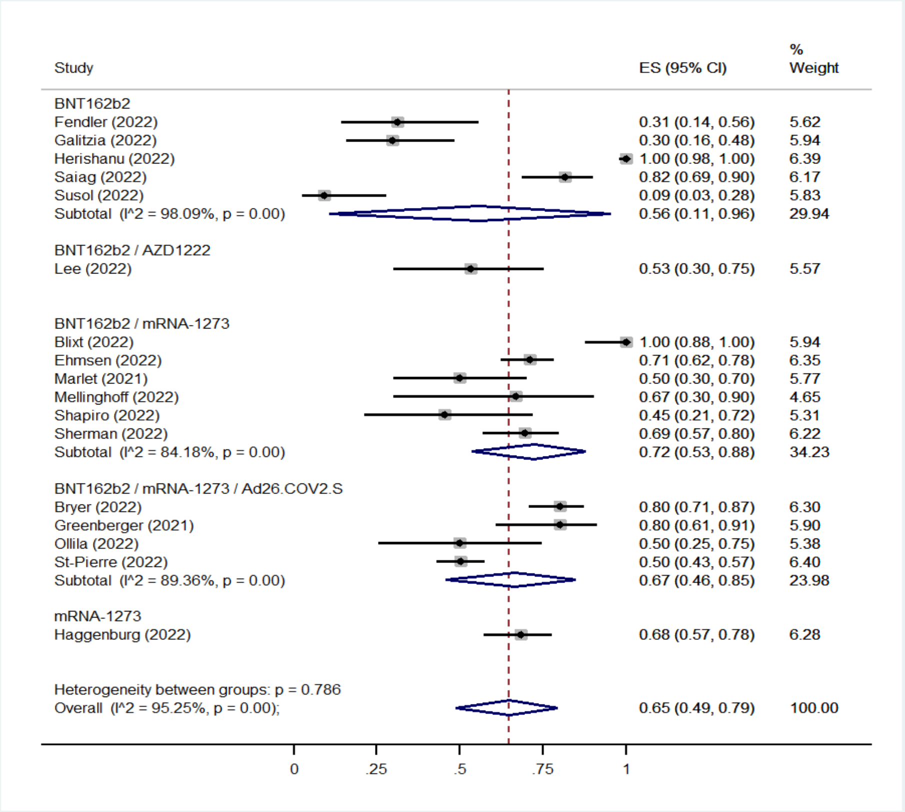

Figure S47. Effect size of immune seroconversion rates of patients with chronic lymphocytic leukemia after third vaccine dose.

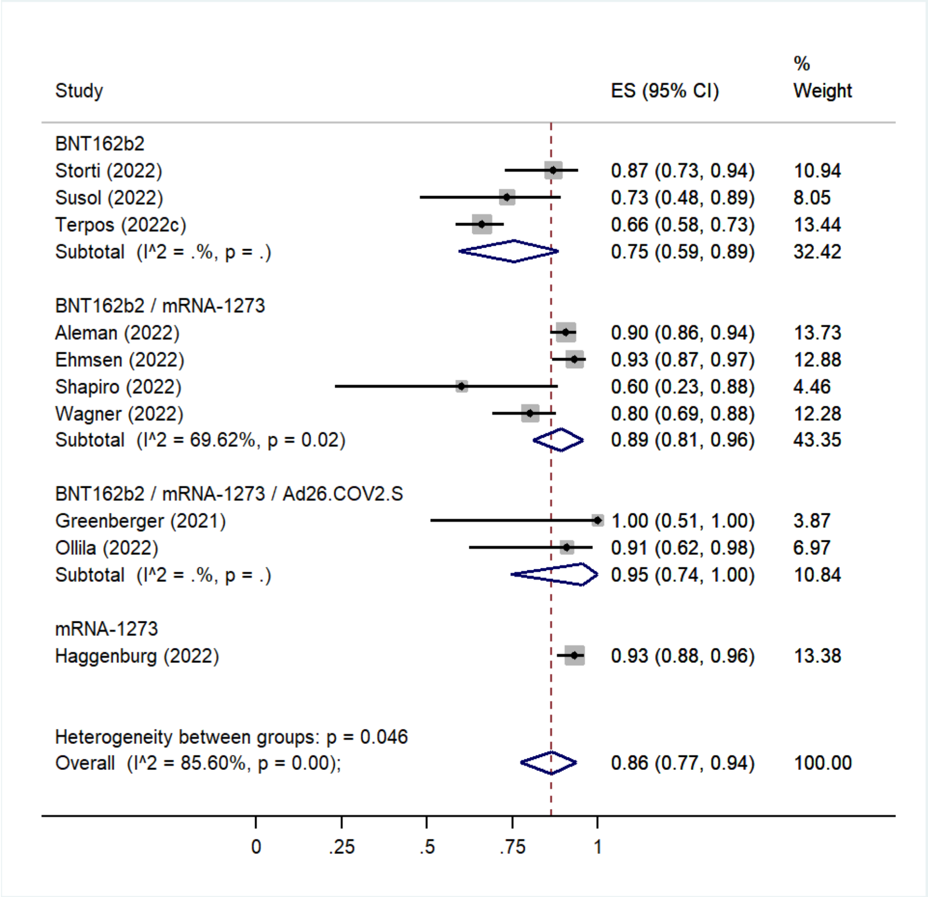

Figure S48. Effect Size of immune seroconversion rates of patients with multiple myeloma after third vaccine dose.

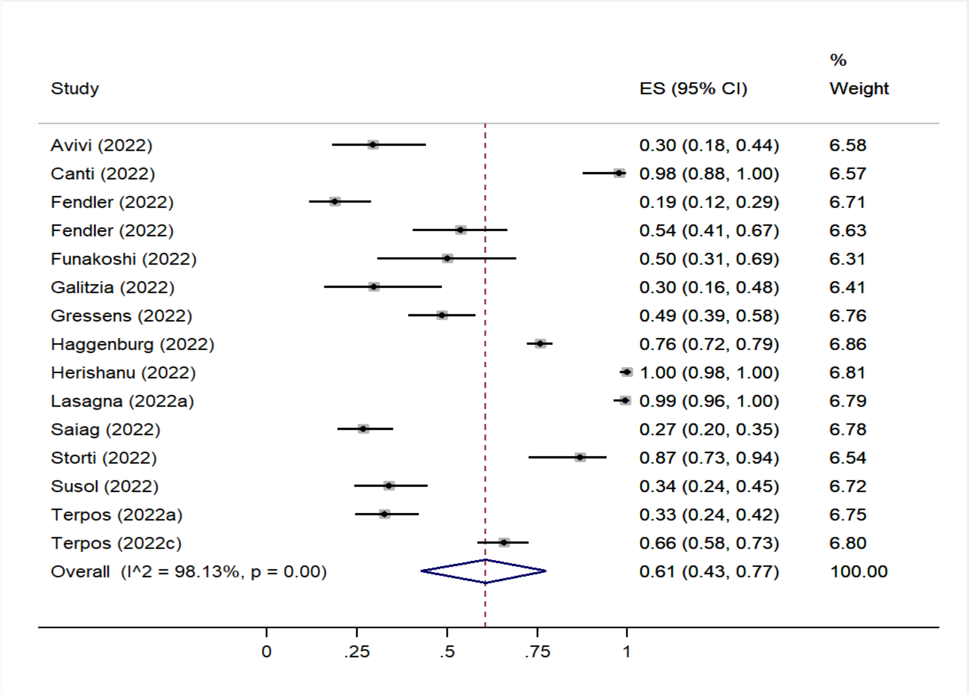

Figure S49. Effect Size for immune seroconversion rates of patients with hematological malignancies who received BNT162B2 booster dose.

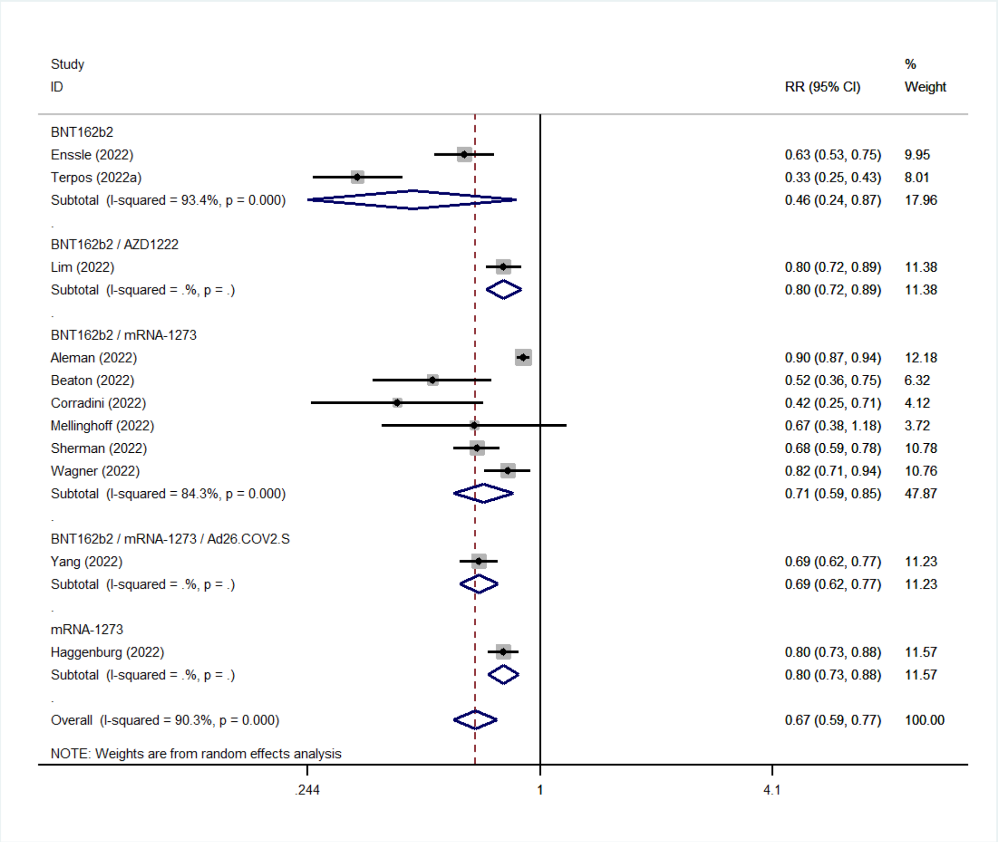

Figure S50. Relative Risk of immune seroconversion rates of patients with hematological malignancies who received a BNT162B2 booster vaccine dose.

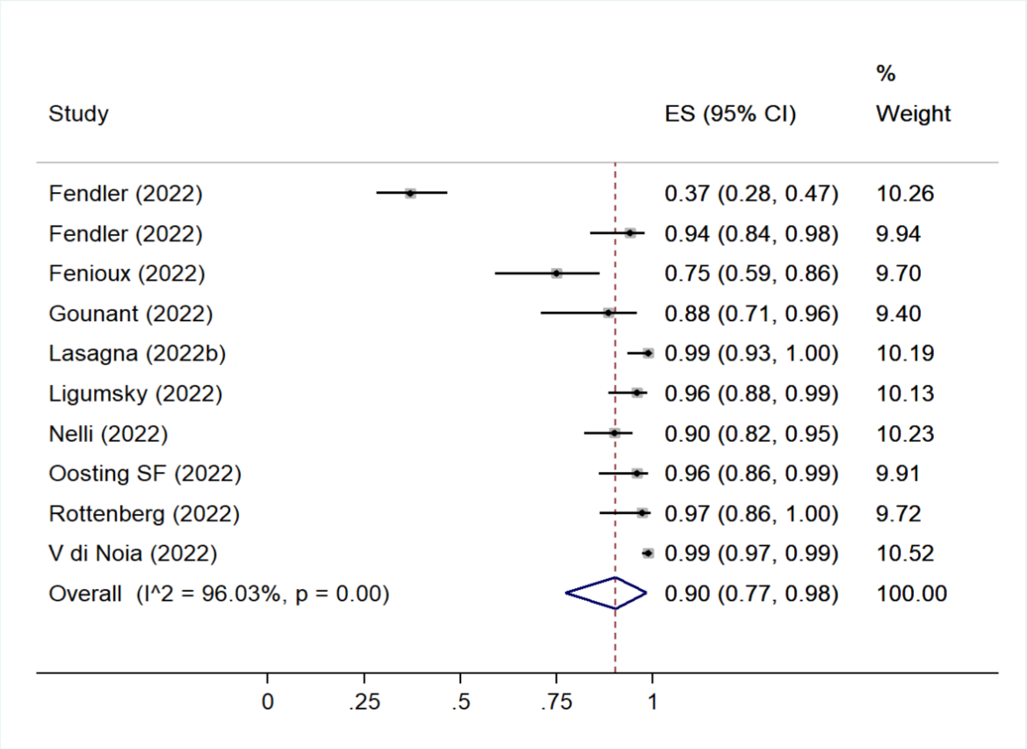

Figure S51. Effect Size of immune seroconversion rates of patients with solid tumors who received a BNT162B2 booster vaccine dose.
