# Supplementary material for: Adult Patients with Cancer Have Impaired Humoral Responses to Complete and Booster COVID-19 Vaccination, Especially Those with Hematologic Cancer on Active Treatment: A Systematic Review and Meta-Analysis
Source: Cancers (Basel). 2023 Apr 12;15(8):2266. doi: 10.3390/cancers15082266 (PMC10136728; doi:10.3390/cancers15082266)
Supplement: Supplementary file 1 [file cancers-15-02266-s001.zip › cancers-2252272-Suppl Table 1-done.pdf]

cancers

MDPI

Newcastle-Ottawa Assessment Scale

Date; Author	Selection				Comparability	Outcome			Total
	Representativeness of the exposed cohort	Selection of the non- exposed cohort	Ascertainment of exposure	Outcome of interest not present at start of study		Assessment of outcome	Adequacy of duration of follow up	Adequacy of completeness of follow up	
Addeo et al. (2021)	+	-	+	+	--	+	+	+	6
Thakkar et al. (2021)	+	-	+	+	--	+	+	_	5
Palich et al. (2021)	+	-	-	+	--	+	+	+	5
Massarwech et al.(2021)	+	+	-	-	++	+	+	-	6
Chowdhurry et al. (2021)	+	-	+	+	--	+	+	+	6
Harrington et al. (2021)	+	-	+	+	--	+	+	+	6
Diefenbach et al. (2021)	+	+	+	+	+-	+	+	+	8
Tzarfati et al. (2021)	+	+	-	+	++	-	+	+	7
Maneikis et al. (2021)	+	+	+	+	++	+	+	+	9
Monin et al. (2021)	+	_	+	+	++	+	+	+	8
Agha et al. (2021)	+	-	-	+	--	+	+	+	6
Bird et al. (2021)	+	-	-	+	+-	+	+	-	5
Pimpinelli et al. (2021)	-	+	+	_	++	+	-	-	5
Iacono et al. (2021)	-	-	+	+	+-	+	+	+	7
Terpos et al. (2021)	+	+	+	+	++	+	+	+	9
Lim et al, (2021)	+	-	+	+	--	+	+	+	6
Gavriatopoulou et al. (2021)	+	+	+	+	++	+	+	+	9

**Herishanu et al. (2021)**	+	+	+	+	++	+	+	+	9
**Terpos et al. (2021)**	+	+	+	+	++	+	+	+	9
**Zagouri et al. (2021)**	+	+	+	-	++	-	+	+	7
**Stampfer et al. (2021)**	+	+	+	+	++	+	+	+	9
**Terpos et al. (2021)**	+	+	+	+	++	+	+	+	9
**Avivi et al. (2021)**	+	+	+	+	--	+	+	+	7
**Nelli et al. (2021)**	+	+	-	-	++	+	-	+	6
**Peeters et al. (2021)**	+	+	+	+	--	+	+	+	7
**Lasagna et al. (2021)**	+	-	+	+	-+	+	+	+	7
**Easdale et al. (2021)**	+	-	+	+	--	+	+	+	6
**Dhakal et al. (2021)**	+	-	+	+	--	+	+	+	6
**Ram et al. (2021)**	+	-	+	+	--	+	+	+	6
**Redjoul et al. (2021)**	+	-	+	+	--	+	+	+	6
**Mairhofer et al. (2021)**	+	+	+	+	++	+	+	+	9
**Perry et al. (2021)**	+	+	+	+	++	+	+	+	9
**Tamari et al. (2021)**	+	-	+	+	--	+	+	+	6
**Ollila et al. (2021)**	+	-	+	+	--	+	+	+	6
**Fendler et al. (2021)**	+	-	+	+	-+	+	-	+	6
**Kastritis et al. (2021)**	+	+	+	+	++	+	+	+	9
**Ehmsen et al. (2021)**	+	-	+	+	--	+	+	+	6
**Chung et al. (2021)**	+	+	+	+	++	+	+	+	9

**Gurion et al. (2021)**	+	+	+	+	-+	+	+	-	7
**Roeker et al. (2021)**	+	-	+	+	--	+	+	+	6
**Del Poeta et al. (2021)**	+	-	+	+	--	+	+	+	6
**Benjamini et al. (2021)**	+	-	+	+	--	+	+	+	6
**Parry et al. (2021)**	+	+	+	+	++	+	+	+	9
**Malard et al. (2021)**	+	-	+	+	--	+	+	+	6
**Benda et al. (2021)**	+	-	+	+	--	+	+	+	6
**Oekelen et al. (2021)**	+	-	+	+	--	+	+	+	6
**Greenberger et al. (2021)**	+	-	+	+	--	+	+	+	6
**Cavanna et al. (2021)**	+	+	+	+	++	+	+	+	9
**Jurgens et al. (2021)**	+	-	+	+	+-	+	+	+	7
**Ghione et al. (2021)**	+	-	+	+	++	+	+	+	8
**Ghandili et al (2021)**	+	+	+	+	++	+	+	-	8
**Gastinne et al (2021)**	+	+	+	+	--	+	+	+	6
**Le Bourgeois et al. (2021)**	+	-	+	+	--	+	+	+	6
**Marchesi et al. (2021)**	+	-	+	+	++	+	+	+	8
**Molica et al. (2021)**	+	-	+	+	--	+	+	+	6
**Linardou et al. (2021)**	+	+	+	+	++	+	+	+	9
**Mair et al. (2021)**	+	-	+	+	++	+	+	+	8
**Jimenez et al. (2021)**	+	-	+	+	--	+	+	+	6

**Zeng et al. (2021)**	+	-	+	+	++	+	+	+	8
**Lysk et al. (2021)**	+	-	+	+	--	+	+	+	6
**Shah et al. (2021)**	+	-	+	+	--	+	+	+	6
**Greenberger et al. (2021**	+	-	+	+	--	+	+	+	6
**Singer et al. (2021)**	+	-	+	+	--	+	+	+	6
**Chumsri et al. (2021)**	+	+	+	+	++	+	+	+	9
**Re et al. (2021)**	+	-	+	+	--	+	+	+	6
**Fox et al (2021)**	+	-	+	+	--	+	+	+	6
**Di Noia et al. (2021)**	+	-	+	+	--	+	+	+	6
Shroff et al. (2021)	-	-	+	+	--	+	-	+	4
Greenberger et al. (2021)	-	-	+	-	--	+	+	+	4
**Herishanu et al. (2022)**	+	-	+	+	--	+	+	+	6
**Shapiro at al. (2022)**	+	-	+	+	--	+	+	+	6
**Marlet et al. (2021)**	-	-	+	-	--	+	+	+	4
**Saiag et al. (2022)**	+	-	+	+	--	+	+	+	6
**Schubert et al.(2022)**	-	-	+	+	--	+	+	-	4
**Ollila et al. (2022)**	+	-	+	-	--	+	+	+	5
**Re et al. (2022)**	-	-	+	+	--	+	+	+	5
**Kimura et al. (2022)**	+	-	+	+	--	+	+	+	6
**Mair et al.(2022b)**	+	-	+	-	--	+	-	+	4
**Gössi et al. (2022)**	+	-	+	+	--	+	-	+	5
**Lasagna et al. (2022a)**	+	-	+	+	--	+	+	+	6
**Canti et al.(2022)**	+	-	+	+	--	+	+	+	6
**Debie et al.(2022)**	+	-	+	+	--	+	+	+	6
**Reimann et al (2022)**	+	-	+	+	--	+	+	+	6

**V. Di Noia et al. (2022)**	+	-	+	-	--	+	+	+	5
**Nelli et al. (2022)**	+	-	+	+	--	+	+	+	6
**Terpos et al. (2022c)**	+	-	+	+	--	+	+	+	6
**Thompson et al. (2022)**	+	-	+	+	--	+	+	-	5
**Galitzia et al. (2022)**	+	-	+	_	--	+	+	-	4
**Funakoshi et al. (2022)**	+	-	+	-	--	+	-	+	4
**Gounant et al. (2022)**	-	-	+	-	--	+	-	-	2
**Fendler et al. (2022)**	+	-	+	-	--	+	-	+	4
**Bilal Abid et al. (2022)**	+	-	+	-	--	+	_	+	4
**Ehmsen et al. (2022)**	+	-	+	-	--	+	+	+	5
**Oosting SF et al. (2022)**	-	-	+	+	--	+	+	+	5
**Einarsdottir et al. (2022)**	+	-	+	+	--	+	+	+	6
**Bagacean (2022)**	-	-	+	-	--	+	+	+	4
**Rottenberg (2021)**	+	-	+	-	--	+	-	+	4
**Fenioux (2022)**	+	-	+	+	--	+	+	+	6
**Gressens (2022)**	+	-	+	-	--	+	-	+	4
**Avivi (2022)**	+	+	+	+	++	+	+	+	9
**Šušol (2022)**									
**Storti (2022)**	-	-	+	-	--	+	-	+	3
**Bryer (2022)**	+	-	+	-	--	+	+	+	5

**Chang (2022)**	+	-	+	+	--	+	-	+	5
**St-Pierre (2022)**	+	-	+	+	--	+	-	+	5
**Lee (2022)**	+	-	+	+	--	+	+	+	6
**Zeng (2022)**	+	-	+	-	--	+	+	+	5
**Blixt (2022)**	+	-	+	+	--	+	+	-	5
**Lasagna (2022b)**	+	-	+	+	--	+	+	+	6
