# Supplementary material for: Adult Patients with Cancer Have Impaired Humoral Responses to Complete and Booster COVID-19 Vaccination, Especially Those with Hematologic Cancer on Active Treatment: A Systematic Review and Meta-Analysis
Source: Cancers (Basel). 2023 Apr 12;15(8):2266. doi: 10.3390/cancers15082266 (PMC10136728; doi:10.3390/cancers15082266)
Supplement: Supplementary file 1 [file cancers-15-02266-s001.zip › cancers-2252272-Suppl Table 2-done.pdf]

cancers

MDPI

Newcastle-Ottawa Assessment Scale

Date; Author	Selection				Comparability	Outcome			Total
	Is the case definition adequate?	Representativeness of the cases	Selection of Controls	Definition of Controls		Ascertainment of exposure	Same method of ascertainment for cases and controls	Non-Response rate	
Terpos et al.(2022a)	+	+	+	+	++	+	+	+	9
Terpos et al. (2022b)	+	+	+	+	+ +	+	+	+	9
Di Giacomo et al. (2022)	+	+	-	+	--	+	+	+	6
Yang et al.(2022)	+	+	-	+	--	+	+	+	6
Mair et al.(2022a)	+	+	-	+	--	+	+	+	6
Lim et al.(2022)	+	+	-	+	--	+	+	+	6
Corradini et al. (2022)	+	+	+	+	++	+	+	+	9
Ligumsky et al.(2022)	+	+	-	+	--	+	+	+	6
Mellinghoff et al.(2022)	+	+	-	+	--	+	+	+	6
Beaton et al. (2022)	+	+	+	+	--	+	+	+	7
Wagner et al. (2022)	+	+	_	+	+-	+	+	+	7
Aleman et al.(2022)	+	+	+	+	+-	+	+	+	8
Enssle et al. (2022)	+	+	+	+	++	+	+	+	9
Yifei et al. (2022)	+	+	+	+	--	+	+	+	7
Zagouri et al. (2022)	+	+	+	+	+-	+	+	+	8
Su et al. (2022)	+	+	+	+	+-	+	+	-	7
Haggenburg et al.(2022)	+	+	+	+	--	+	+	+	7

Sherman et al. (2022)	+	+	+	+	--	+	+	+	7
